# Structure–Function Analysis of an Understudied Type of LPMO with Unique Redox Properties and Substrate Specificity

**DOI:** 10.1021/acscatal.5c03003

**Published:** 2025-06-06

**Authors:** Kelsi R. Hall, Synnøve Elisa Rønnekleiv, Alfonso Gautieri, Hedda Lilleås, Rannei Skaali, Lukas Rieder, Andrea Nikoline Englund, Eirin Landsem, Tom Z. Emrich-Mills, Iván Ayuso-Fernández, Åsmund Kjendseth Røhr, Morten Sørlie, Vincent G. H. Eijsink

**Affiliations:** † Faculty of Chemistry, Biotechnology and Food Science, Norwegian University of Life Sciences (NMBU), 1432 Ås, Norway; ‡ Biomolecular Engineering Lab, Dipartimento di Elettronica, Informazione e Bioingegneria, Politecnico di Milano, Piazza Leonardo da Vinci 32, 20133 Milano, Italy; § Institute for Molecular Biotechnology, 27253 Graz University of Technology, 8010 Graz, Austria; ∥ Biomolecular Interaction Centre and School of Biological Sciences, 2496 University of Canterbury, PO Box 4800, Christchurch 8140, New Zealand

**Keywords:** enzyme catalysis, copper, LPMO, substrate specificity, chitin, peroxygenase, hole hopping

## Abstract

Lytic polysaccharide monooxygenases (LPMOs) are important biotechnological tools due to their ability to activate C–H bonds in recalcitrant polysaccharides. To-date, most research has focused on LPMOs from the AA9 and AA10 families, while LPMOs from the AA11 family have not received the same attention since their classification almost a decade ago, despite their wide abundance in fungi. Previous studies have shown that *Af*AA11B from Aspergillus fumigatus has exceptionally high oxidase activity, low reduction potential and the ability to degrade soluble chitooligomers. To better understand the catalytic capabilities of *Af*AA11B, its crystal structure was solved, revealing a unique flexible surface loop that mediates activity on soluble substrates, as shown by molecular dynamics simulations and mutagenesis. Mutation of an active site Glu residue to a Gln, Asp or Asn showed that this residue is crucial in controlling the low reduction potential and high oxidase activity of *Af*AA11B. The impact of these mutations on copper reactivity aligned well with results obtained for an AA9 LPMO, which naturally has a Gln in this position. However, the impact of these mutations on the productive peroxygenase reaction, measured using an electrochemical hydrogen peroxide sensor, and on protective hole hopping mechanisms, measured using stopped-flow ultraviolet–visible (UV–vis) spectrophotometry, differed from the AA9 LPMO. This shows that the impact of this Glu/Gln residue is dependent on additional structural or dynamic differences between the LPMOs. Despite the presence of several tryptophan residues in the protein core, the hole hopping studies revealed formation of only a tyrosyl feature with a lifespan distinct from similar features detected in other LPMOs, further highlighting the unique properties of *Af*AA11B.

## Graphical abstract

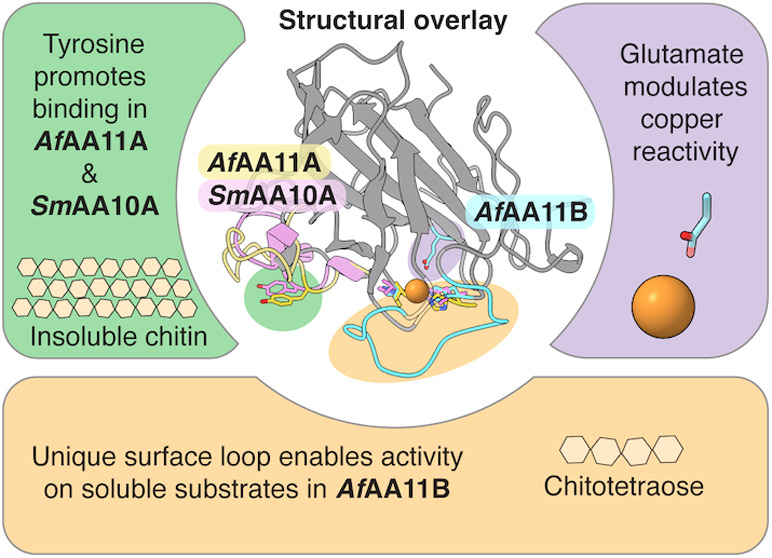

## Introduction

The catalytic capabilities of lytic polysaccharide monooxygenases (LPMOs) were first discovered in 2010 ^1^ and LPMOs have since garnered significant attention due to their unique catalytic power and biotechnological applications. These enzymes play a pivotal role in biomass degradation, through activation of C–H bonds at the C1 or C4 position of scissile glycosidic bonds, allowing the degradation of recalcitrant substrates such as crystalline cellulose or chitin. LPMOs are used in various biotechnological applications, in particular for boosting the efficiency of hydrolytic enzyme systems^1–4^ to enable more sustainable conversion of lignocellulosic biomass into value-added products.^5–7^ LPMOs belong to the broader class of auxiliary activity (AA) enzymes and as of 2024 are classified into eight of the 17 AA families (AA9–AA11 and AA13–AA17).^8^ Within these eight LPMO families, activity has been demonstrated on a range of substrates including cellulose,^3,4,9^ chitin,^1^ hemicelluloses,^10–12^ and starch.^13,14^ Roles in microbial pathogenesis^15–17^ and cellular development^18,19^ have also been demonstrated, illustrating the wide impact and potential applications of these enzymes.

While fungal and bacterial LPMOs belonging to the large AA9 and AA10 families, respectively, have been studied intensively^20,21^ much less is known about LPMOs belonging to the other families. Of these other families, the AA11 family, with only five (partially) characterized representatives, is of particular interest as this family is relatively large and AA11 LPMOs are the most widespread of all fungal LPMOs.^22^ In terms of the number of LPMO genes per species, AA11s are, on average less abundant than AA9s, however fungal species with more than 20 AA11 genes have been described.^22^ The AA11 family was first classified in 2014 ^23^ with reported activity on chitin. The limited functional data accumulated so far indicates that some AA11 LPMOs indeed act on polymeric chitin, whereas other family members, remarkably, seem to prefer soluble chitin fragments as substrates.^23–26^ Structural information is only available for the catalytic domains of *Ao*AA11 ^23^ from Aspergillus oryzae and *Af*AA11A^25^ from Aspergillus fumigatus. Of note, and of relevance for the work presented below, the *Ao*AA11 structure is incomplete, lacking density for 30 of the in total 216 residues.

The genome of A. fumigatus encodes three AA11 LPMOs that are coexpressed.^27^ *Af*AA11A, a single domain AA11 for which a complete crystal structure exists, shows structural and functional features associated with activity on chitin. Accordingly, it has been shown that this enzyme enhances the solubilization of α-chitin and β-chitin by chitinases such as *Sm*ChiC, similar to what has been observed for bacterial chitin-active LPMOs such as the archetypal *Sm*AA10A, also known as CBP21.^25^ In contrast, *Af*AA11B has shown little activity on chitin, while exhibiting high activity on soluble chito-oligosaccharides.^24^ *Af*AA11B shows only 39.6 and 37.5% sequence identity with *Af*AA11A and *Af*AA11C, while it has 72.6% sequence identity with *Ao*AA11. While *Ao*AA11 is reported to be active on chitin,^23^ its activity has not been quantified and it is thus not clear if this activity is high (*Af*AA11A-like) or low (*Af*AA11B-like). Of note, in both *Af*AA11B and *Ao*AA11, the catalytic domain is followed by an extended linker of low sequence complexity and a C-terminal X278 domain with unknown function.

Adding another unique feature to *Af*AA11B, this enzyme has the lowest reduction potential and highest oxidase activity of any characterized wild-type LPMO.^24^ These characteristics could make *Af*AA11B of particular interest to biotechnologists, who could potentially harness its unique redox properties in novel applications. The histidine brace is a unifying feature of all LPMOs, whereby two histidine residues in the LPMO coordinate the copper ion found in the active site.^4^ However, it alone does not give LPMOs their oxidizing power: other LPMO-like proteins have this histidine brace but lack any reported peroxygenase activity,^28–30^ showing that residues beyond the histidine brace, sometimes referred to as the second copper coordination sphere, are crucial for catalysis. Interestingly, the second sphere arrangements in LPMOs vary greatly, with no universally conserved residue present in the second sphere. Therefore, understanding the diversity that exists at these positions is imperative for understanding LPMO catalysis.

Studies of LPMO catalysis have been hampered by initial confusion regarding the reaction that these enzymes catalyze. Originally, LPMOs were thought to be monooxygenases (RH + O_2_ + 2e^–^ + 2H^+^ → ROH + H_2_O), but, recently, it has become clear that LPMOs may also act as peroxygenases, using hydrogen peroxide as the cosubstrate.^31–38^ It has been shown that in typical reductant-driven LPMO reactions (“monooxygenase” conditions), the reaction may be limited by slow *in situ* generation of the hydrogen peroxide cosubstrate, resulting from abiotic oxidation of the reductant and an off-pathway reductant oxidase activity of the LPMO.^39–41^ In contrast, the peroxygenase reaction requires a priming reduction of LPMO-Cu­(II) to LPMO-Cu­(I) after which the enzyme can catalyze multiple productive peroxygenase reactions, oxidizing its polysaccharide substrate.^38^ It is generally assumed that the productive reaction involves homolytic cleavage of hydrogen peroxide leading to the formation of a Cu­(II)-hydroxide and a hydroxyl radical (HO^•^).^35,38,42,43^ The hydroxyl radical converts the Cu­(II)-hydroxide to a Cu­(II)-oxyl species that is thought to abstract a hydrogen atom from the substrate.^42–45^ Importantly, generation of these highly oxidizing intermediates requires their precise confinement in the enzyme–substrate complex, to avoid off-pathway reactions that might damage the enzyme.^38,46^ Reduced LPMOs that interact with hydrogen peroxide in the absence of substrate are prone to oxidative damage. Computational and mutational studies have shown that in enzyme–substrate complexes, a second sphere glutamine or glutamate residue plays an important role in positioning hydrogen peroxide and confining emerging oxidative species.^42,43,47,48^

Here, by solving the crystal structure of *Af*AA11B, followed by molecular dynamics analysis of enzyme–substrate complexes, we unravel features of this enzyme’s substrate binding surface that were not visible in the incomplete structure of closely related *Ao*AA11, and that may explain why this enzyme prefers soluble substrates. Furthermore, we have studied the effect of mutation on the second sphere glutamate that, based on previous studies^47^, is a crucial determinant of copper redox properties. This glutamate is part of a second-sphere arrangement that is unique for AA11 LPMOs and the results of the mutational work reveal differences between the impact of this residue in *Af*AA11B, compared to well-studied cellulose-active *Nc*AA9C. These findings stress the inadequacy of adopting a “one-size-fits-all” approach when it comes to understanding LPMO catalysis.

## Results and Discussion

### Phylogenetic Analysis

The maximum likelihood phylogenetic tree of the catalytic domains of AA11 LPMOs shows a distinct organization in two major clades (Figure 1). Clade I includes *Af*AA11A, an enzyme with well-documented activity on insoluble chitin and a known ability to potentiate the action of chitinases.^25^ In contrast, *Af*AA11B, with little activity on insoluble chitin and high activity on soluble chitin oligomers, clusters together with *Ao*AA11 in a subclade of clade II. The robust topology of the tree highlights the phylogenetic distance and ancestral diversification between the main two proteins discussed in this study, *Af*AA11A^25^ and *Af*AA11B.

**Figure 1:**
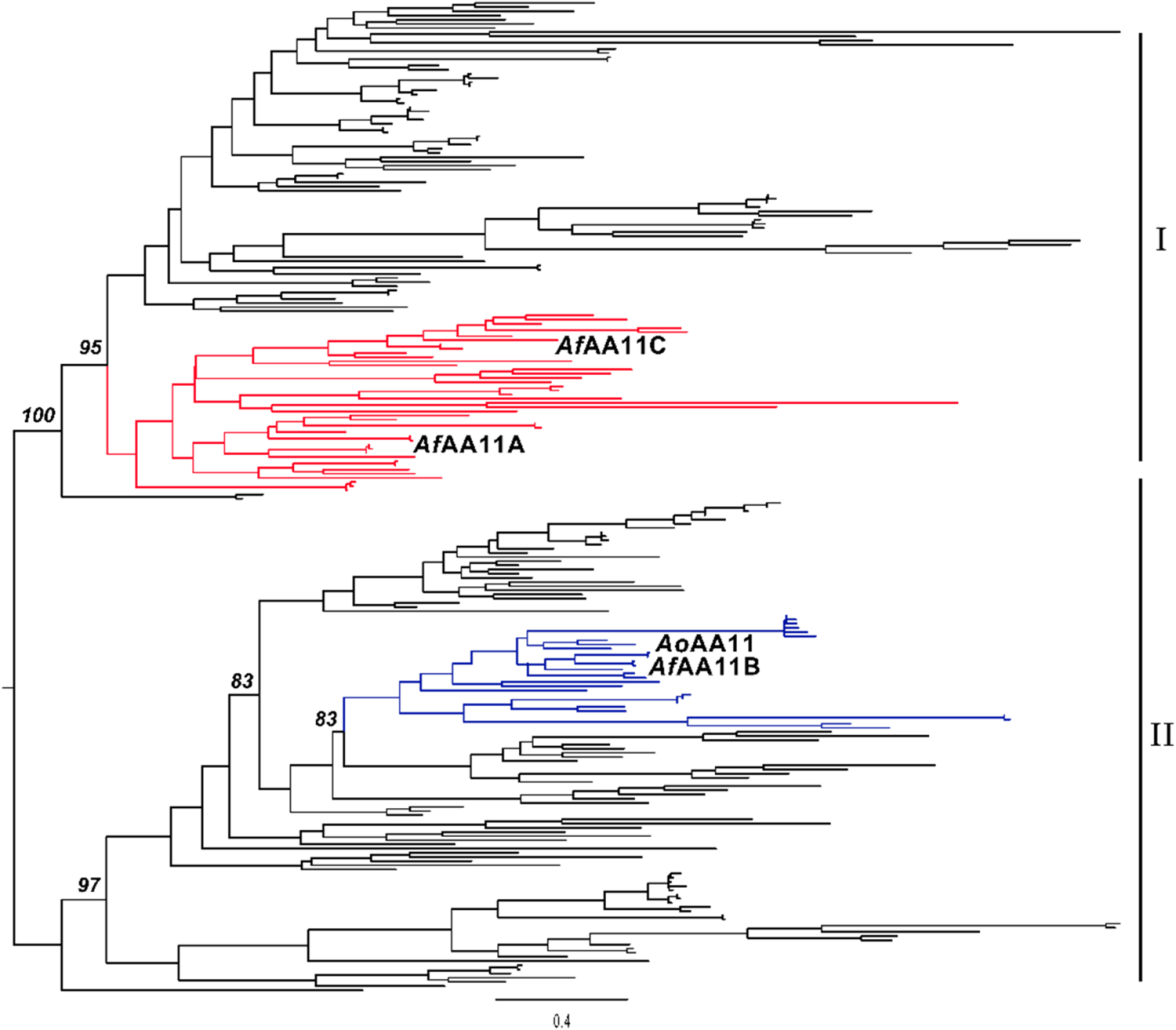
Simplified maximum likelihood tree of the catalytic domains of AA11 LPMOs. The subclades containing the LPMOs discussed in this study are highlighted in red (*Af*AA11C and *Af*AA11A) or blue (*Ao*AA11 and *Af*AA11B). Bootstrap values for the main diversification events are indicated. For a full phylogenetic tree containing the accession codes of all 238 proteins and all bootstrap values, see Figure S1.

### Crystal Structure of *Af*AA11B and the Structural Basis of Its Substrate Specificity

A gene fragment encoding the catalytic domain of *Af*AA11B (residues 1–219) without its native signal peptide was cloned into the pBSY3Z-P*_DAS2_* vector. The assembled construct allows tight transcriptional control of the LPMO gene by methanol induction and secretion of the mature LPMO to the culture broth *via* the Ost1 signal peptide, using Komagataella phaffii (reclassified from Pichia pastoris) as the microbial production host. Prior to setting up crystal screens, the mature protein was de-*N*-glycosylated using *Ef*Endo18A (Figure S2). Crystals were obtained in conditions containing 0.1 M Tris, pH 8.5, 2 M ammonium sulfate (Figure S3) and the structure was solved using X-ray crystallography. An almost complete structure at 1.26 Å resolution was obtained, with a copper ion bound in the histidine brace. Residues 152–172, likely comprising a mobile surface loop (see below) could not be modeled in the electron density maps. Diffraction data and refinement statistics are shown in Table 1.

**Table 1:** Diffraction Data and Refinement Statistics for the *Af*AA11B Crystal Structure

crystal data		refinement statistics	
space group	*P*2_1_2_1_2_1_	*R*_crystal_ ^c^	0.1645
cell dimensions: *a*, *b*, *c* (Å) α, β, γ (°)	46.332 53.655 70.929 90 90 90	*R*_free_ ^d^	0.1958

data collection		validation	
beamline	ID30A-3 (ESRF)	RMS (bonds)	0.008
wavelength (Å)	0.873	RMS (angles)	1.00
resolution (Å)	35.46–1.26 (1.29–1.26)^a^	Ramachandran favored (%)	96.91
number unique reflections	47610 (4611)^a^	Ramachandran allowed (%)	3.09
completeness	98.4 (98.7)^a^	Ramachandran outliers (%)	0.00
multiplicity	4.2 (3.9)^a^	Rotamer outliers (%)	1.74
CC half	1 (0.331)^a^	Added waters	230
mean (*I*/σ*I*)	7.96 (0.62)^a^	Added ligands	1 Cu, 1 ascorbate, 1 glucose, 6 SO_4_
*R*_merge_ ^b^	0.053 (1.405)^a^		

a Values in parentheses are for the outer-shell.

b *R*_merge_ = ∑|*I* – ⟨*I*⟩ |/∑*I*.

c *R*_crystal_ = ∑(|*F*_obs_ – *F*_calc_|)/∑|*F*_obs_|.

d *R*_free_ is calculated from a randomly chosen 5% sample of all unique reflections not used in the refinement.

The *Af*AA11B structure shows a typical LPMO fold, with a pyramid-like shape and a canonical immunoglobin-like β-sandwich fold with several loops and two small helices (Figure 2A). The β-sandwich is comprised of two β-sheets, one consisting of four antiparallel β-strands (S3, S4, S6, S7) and a smaller one consisting of two antiparallel β-strands (S2 and S5). Other secondary structure elements include two additional β-strands (S1 and S8) and two short α-helices, where the latter occur at the edges of the substrate binding surface, which is made up of a series of loops. Although little is known about the structural determinants of the substrate specificity of LPMOs, functional diversity in LPMOs is associated with variation in the nature (*e.g.*, hydrophobicity, charge) and shape of the substrate binding surfaces (Figure S4). The substrate binding surface of *Af*AA11B shows two conspicuous features that could relate to its remarkable substrate specificity (Figure 2B). First, *Af*AA11B lacks an exposed tyrosine residue that is found on the substrate binding surface of *Af*AA11A (Y25)^25^ and other chitin-active LPMOs such as *Sm*AA10A (Y27) (Figure 2B).^49^ Previous research has demonstrated the importance of this residue for chitin binding.^49–51^ Second, the structure of *Af*AA11B reveals one of the two loops that was not visible in the previously solved structure of *Ao*AA11 that may affect the substrate binding surface (Figure 2). *Af*AA11A and other LPMOs active on insoluble chitin (*e.g.*, *Sm*AA10A) lack this loop region, which is referred to as the 95–110 loop below.

**Figure 2:**
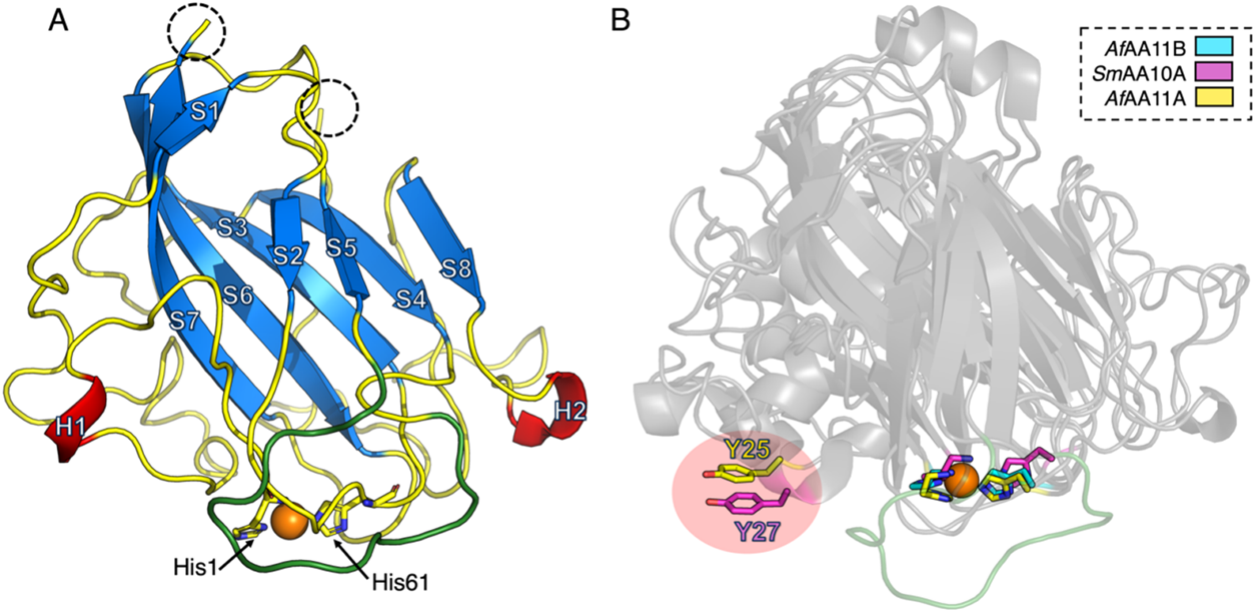
Structure of *Af*AA11B with comparison to chitin-active LPMOs. (A) Structure of the catalytic domain of *Af*AA11B with the bound copper ion shown as an orange sphere and the histidine brace (His1 and His61) shown as sticks with the carbons colored in yellow. Helical regions are shown in red and numbered (H1–H2), β-strands are shown in blue and numbered (S1–8) and loop regions are shown in yellow, except for one loop region of interest (residues 95–110) that is colored in green. The start and end of the loop region at the top of the structure that could not be modeled are labeled by dotted black circles (residues 152–172). (B) Structures of *Af*AA11B (blue), *Af*AA11A (yellow; PDB: 7P3U) and *Sm*AA10A (pink; PDB: 2BEM) are shown, with the histidine brace and other residues of interest colored. The copper ion from *Af*AA11B is shown as an orange sphere. Tyrosine residues predicted to be involved in binding to insoluble chitin are indicated in the red circle. This residue is notably missing in *Af*AA11B. *Af*AA11B contains an extra loop region comprised of 15 residues, indicated in green, which may prevent the LPMO from binding to insoluble chitin. Of note, the highlighted tyrosine residue is also present in the third AA11 of A. fumigatus, *Af*AA11C.

Structural alignment using the Dali server^52^ was performed for *Af*AA11B and all available structures for the auxiliary activity families 9–11 and 13–17 in the CAZy database (*n* = 53).^8^ As expected, LPMOs from the same families largely clustered together with the three structures from the AA11 LPMO family forming a distinct cluster (Figure S5). *Af*AA11B shared the highest structural homology with *Ao*AA11 (PDB: 4MAH) with a Z-score of 31.5 and a root-mean-square deviation (RMSD) score of 2.0 over 186 residues, with these proteins sharing 75% sequence identity. Structurally, the AA11 family seems most closely related to the AA14 family followed by the AA9 family. Unfortunately, only a single structure is available for the AA14 family.^53^ Solving more structures in the AA11 and AA14 families may provide insights into the connection between these two families and help unravel the enigmatic substrate specificities of enzymes in these families.^24,54^

Several attempts were made to predict the structure of the unresolved 152–172 loop region, but this region could not be modeled using AlphaFold,^55^ RoseTTAFold,^56^ SwissModel^57^ or RosettaRemodel.^58^ While AlphaFold, RosettaFold and SwissModel predicted a disordered structure (Figure S6A), RosettaRemodel predicted the presence of α helices (Figure S6B). Molecular dynamics simulations were then done using input models from AlphaFold and RosettaRemodel to assess the structure of the loop. Taken together, these analyses did not provide a defined structure for this region and led to the overall conclusion that this region is highly flexible (Figure S6C,D). Importantly, none of these analyses provided indications that the 152–172 loop interferes with the catalytic center or the substrate-binding surface. Of note, the MD simulations showed that the 95–110 loop, which we did observe crystallographically, is also flexible (Figure S6C,D).

Models of *Af*AA11B and *Af*AA11A in complex with crystalline β-chitin and chitooligomers were generated as outlined in Figure S7. Simulations of the interaction of *Af*AA11A or *Af*AA11B with β-chitin suggested stable binding of *Af*AA11A, whereas for *Af*AA11B the histidine brace moved away from the chitin surface (Figure S8). While more thorough analyses are needed to understand how *Af*AA11B may interact with chitin, it is clear that this interaction is affected by its unique 95–110 loop (Figure S8). In 200 ns MD simulations of *Af*AA11A, which is not active on soluble substrates, in complex with chitobiose, chitotetraose and chitohexaose (see Figure S7 for details) only the hexaose, occupying subsites −4 to +2, showed a clear tendency to stay bound to the enzyme (Figure S9; binding was also observed for one of three runs with a tetramer bound to subsites −3 to +1). Similar MD experiments with *Af*AA11B showed a strong tendency for both the hexaose (bound to subsites −4 to +2) and, importantly, the tetraose when bound to subsites −2 to +2, to stay bound (Figure S9). Binding of a chitotetraose to subsites – 2 to +2 correlates very well with the observed ability of *Af*AA11B to convert chitotetraose to a native and an oxidized dimer.

Importantly, in the simulations with *Af*AA11B and chitotetraose, the otherwise flexible 95–110 loop (Figure 2A) adopted a fixed configuration stabilized by a hydrogen bond between Asn99 and the sugar bound in subsite +1 (Figure 3).

**Figure 3:**
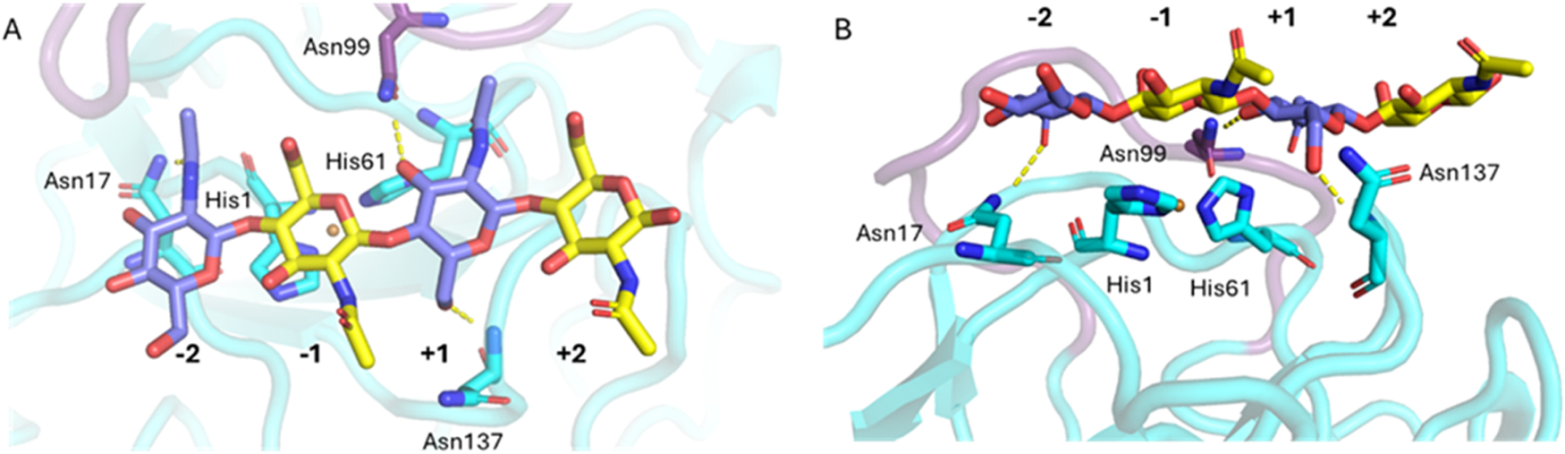
Molecular model of *Af*AA11B in complex with chitotetraose. (A) Top-view and (B) Side-view of the complex obtained after 200 ns of MD simulation starting from the model obtained by trimming the crystalline chitin in the *Af*AA11B-chitin model, leaving only part of the chitin chain that is binding to the catalytic copper site (see Materials and Methods and Figure S7 describing the building of the models). The center of the copper ion is shown as an orange sphere. Oligomer binding is stabilized through strong interactions, including an H-bond between Asn17 and the sugar in subsite −2, an H-bond between the backbone of Asn137 and the sugar in subsite +1, and an H-bond between Asn99, in the 95–110 loop and the sugar bound in subsite +1. Of note, Asn17 is replaced by a threonine in *Af*AA11A. Panel B also shows that the 95–110 loop (in purple, behind the chitin oligomer) would interfere with binding to a flat chitin surface (see Figure S8 for more details).

These observations suggest that the unique 95–110 loop in *Af*AA11B affects substrate binding and promotes activity on soluble substrates. To verify the latter, we generated the N99A mutant of *Af*AA11B. While this mutant showed essentially the same oxidase activity as the wild-type enzyme, showing that the protein was properly folded and bound copper, it had almost completely lost its activity on chitotetraose (Figure S10). This shows that the flexible loop indeed is crucial for activity on chitotetraose and reveals a mechanism by which LPMOs have evolved to act on soluble substrates.

### The Catalytic Center of *Af*AA11B Shows Deviations from Other LPMOs

Looking specifically at the active site, *Af*AA11B shares common LPMO features, including the universally conserved histidine brace coordinating a copper ion (His1 and His61). Despite the observed differences in substrate specificity between AA11 LPMOs, their second spheres are virtually identical, with all three AA11 structures having a tyrosine occupying the proximal axial coordination position of the copper, as well as an asparagine and glutamate (Figure 4A–C). The combination of an axial tyrosine with a glutamate is unique. In AA9 LPMOs such an axial tyrosine occurs with a glutamine instead of a glutamate (Figure 4D), whereas AA10 LPMOs have a combination of an axial phenylalanine with a glutamate (Figure 4E,F). The role of this conserved glutamine/glutamate has been addressed in an AA9 and an AA10 LPMO, and these studies have revealed that it modulates copper reactivity.^47,59^ Furthermore, both structural, mutational and computational studies have shown that this residue plays a role in coordinating (and, perhaps, recruiting) hydrogen peroxide, and in directing the intermediates formed during catalysis toward a productive reaction, thus preventing enzyme damage.^42,43,48^ To investigate the role of this residue in *Af*AA11B, with its unprecedented active site configuration (Figure 4), the glutamate residue in *Af*AA11B at position 139 was mutated to glutamine, aspartate, or asparagine in the full-length enzyme. Thus, we altered the charge of the functional group as well as its distance from the copper and the axial tyrosine. The closest contacts of Glu139 in the wild-type enzyme are 2.8 Å for the tyrosine and 4.5 Å for the copper.

**Figure 4:**
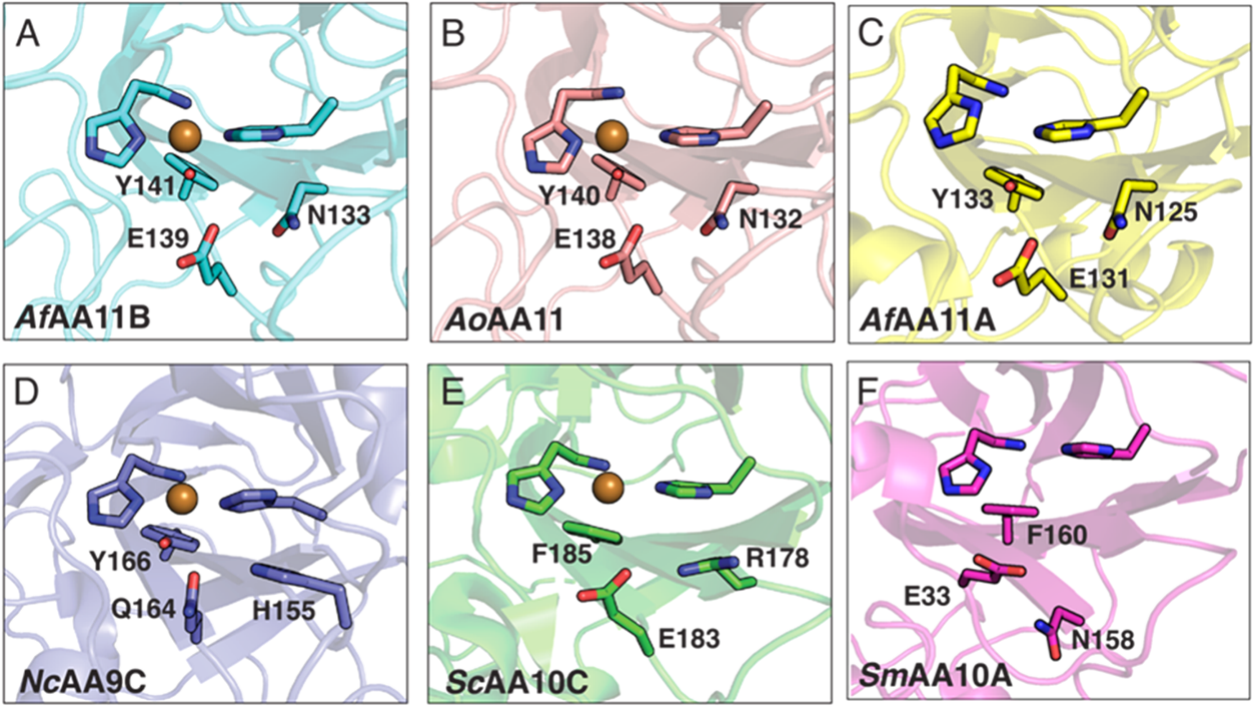
Second sphere residues in the active sites of selected LPMOs. (A) *Af*AA11B, (B) *Ao*AA11; PDB4OPB, (C) *Af*AA11A; PDB 7P3U, (D) *Nc*AA9C; PDB 4D7U, (E) *Sc*AA10C; PDB 4OY7 and (F) *Sm*AA10A; PDB 2BEM. Residues not belonging to the histidine brace are labeled. All numbering is for the mature protein with the N-terminal histidine listed as His1. When it comes to the conserved axial Tyr/Phe (*e.g.*, Y141 in *Af*AA11B in panel (A) or F185 in *Sc*AA10C in panel (E)) and the conserved Glu/Gln (*e.g.*, E139 in *Af*AA11B in panel (A) or Q164 in *Nc*AA9C in panel (D)), the six LPMOs show three different combinations: The AA11s have Tyr-Glu, the AA9s have Tyr-Gln and the two shown AA10s have Phe-Glu. Note that the conserved Glu/Gln has a different position in the sequence of *Sm*AA10A (F; E33) compared to all other shown LPMOs.

To assess for activity in the *Af*AA11B mutants, time course experiments were performed under conditions that are sometimes referred to as “monooxygenase conditions”, in which the reaction is limited by the *in situ* generation of the hydrogen peroxide cosubstrate that results from enzymatic and abiotic oxidation of the reductant. All mutants were less active than WT *Af*AA11B on chitotetraose, exhibiting a three to 5-fold decrease in the initial rate of the reaction (Figure 5A). Interestingly, the reduced activity in these hydrogen peroxide-limited reactions correlates with a three to 5-fold reduction in the oxidase activities, *i.e.*, the ability of the LPMO to produce hydrogen peroxide in the absence of substrate (Figure 5B). The reduced oxidase activity of the mutants correlates with these mutants having higher reduction potentials (Figure 5B). These trends and correlations align well with previously described mutational effects for *Nc*AA9C^47^ and for *Ma*AA10B,^59^ which have second sphere arrangements with a glutamine in this position. In these previous studies the glutamate variants (*Nc*AA9C_Q164E and *Ma*AA10B_Q183E) showed the highest oxidase activities.

**Figure 5:**
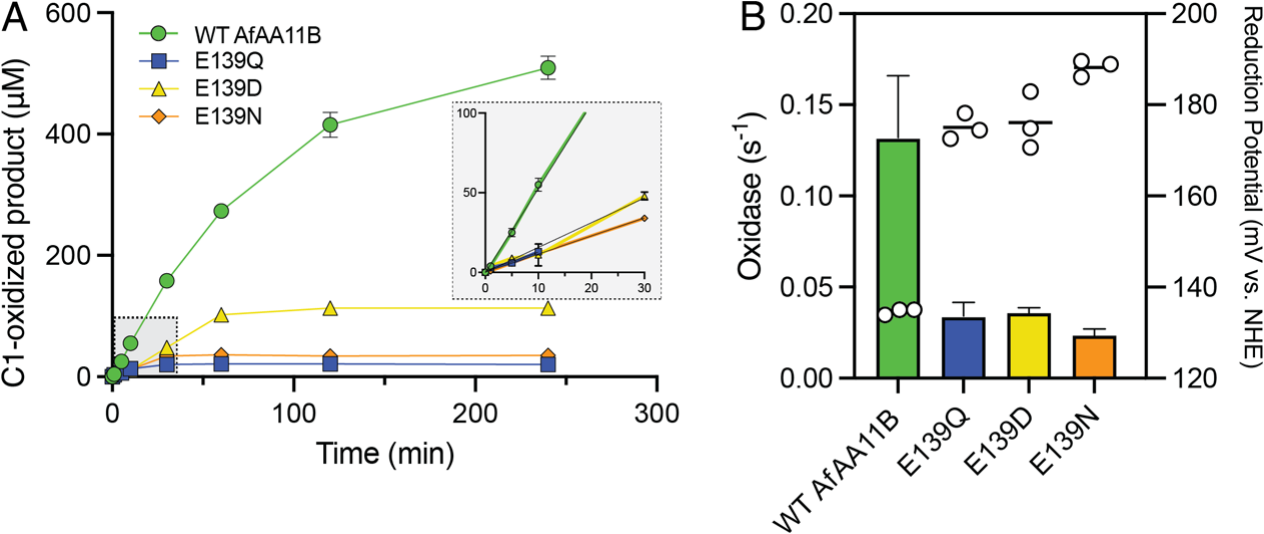
Impact of mutating E139 on LPMO activity in hydrogen peroxide-limited reactions with substrate and on oxidase activity in the absence of substrate. (A) Time course experiments under apparent monooxygenase conditions over 240 min. Reactions contained 1 μM LPMO, 1 mM (GlcNAc)_4_, and 1 mM ascorbate in 50 mM Bis–Tris, pH 6.5, and were performed at 37 °C, 750 rpm. The inset shows the initial rate of reaction for each variant (WT *Af*AA11B, 0.088 ± 0.002 s^–1^; E139Q, 0.019 ± 0.001 s^–1^; E139D, 0.026 ± 0.002 s^–1^; E139N, 0.019 ± 0.001 s^–1^). (B) Oxidase activity *i.e.*, hydrogen peroxide production in the absence of substrate, and measured reduction potentials. The oxidase activity (s^–1^) is plotted as a bar graph on the left-hand *Y*-axis and was measured using a modified Amplex Red/HRP assay at 30 °C. Reactions contained 1 μM LPMO, 1 mM ascorbate and 50 mM Bis–Tris, pH 6.5. The reduction potential (mV *vs* NHE, at pH 6.0) is plotted as a scatter dot plot on the right-hand *Y*-axis and was determined using the redox mediator TMP_red_.^61^ All reported data are the average of 3 independent repeats with the standard deviation reported.

All mutants of *Af*AA11B were less stable, as evidenced by the plateauing product formation curves (Figure 5A). The E139Q mutant was the least stable, inactivating after only 10 min, while the E139D mutant was active over a longer 60 min period before inactivating. These results are different to what was observed previously when studying equivalent variants of *Nc*AA9C,^47^ which, notably has a glutamine in the wild-type enzyme. In this case the glutamine to glutamate mutation clearly made the enzyme prone to off-pathway reactions, leading to earlier inactivation.^47^ It is noteworthy, that, while the impact of the glutamine-glutamate exchange on copper reactivity seems similar between the two enzymes (in both enzymes glutamate gives a lower redox potential and higher oxidase activity; see Figure 5B and ref 47), the two enzymes need different residues in this location to function optimally in the peroxygenase reaction: *Af*AA11B works best with its native glutamate, whereas *Nc*AA9C works best with its native glutamine. The results suggest that AA11 family LPMOs have evolved so a tyrosine-glutamate combination in the active site leads to optimal productive use of available hydrogen peroxide. Comparison of the three *Af*AA11B mutants shows that the negative charge is essential, since the E139Q and E139N mutants perform clearly worse than the E139D mutant. Notably, the better performance of the acidic residues could also relate to the ability of these residues to act as a base during catalysis. LPMOs with a glutamine in this position tend to have a highly conserved histidine nearby, which could also act as a base (*e.g.*, H155 in *Nc*AA9C; Figure 4D). However, several studies have concluded that base catalysis likely is not involved in the peroxygenase reaction, such as a recent experimental study of the pH-dependency of catalysis by an AA9 LPMO^60^ and a theoretical analysis of catalysis in an AA10 LPMO.^42^

To gather further insight into the mutational effects on enzyme performance, we eliminated the influence of the oxidase activity by carrying out time course reactions in the presence of 100 μM exogenous hydrogen peroxide. Such conditions allow the true peroxygenase activity to be monitored, but the high initial level of hydrogen peroxide may lead to enzyme inactivation. Under such conditions the *Af*AA11B reaction is fast (s^–1^ rather than min^–1^ scale), making it difficult to determine the true initial rate. The progress curves (Figure 6A) suggest that WT *Af*AA11B and the E139D mutant are the two fastest variants, in accordance with results discussed above, suggesting that a carboxylic acid group is crucial for enzyme performance. The progress curves depicted in Figure 6A show that the wild-type rapidly consumes all added H_2_O_2_ in a productive reaction, in line with previous observations,^24^ while confirming the notion that the three mutants are less capable of using hydrogen peroxide productively and are thus prone to inactivation. The product levels show that WT *Af*AA11B consumed all added hydrogen peroxide productively, whereas this was not the case for the mutants.

**Figure 6:**
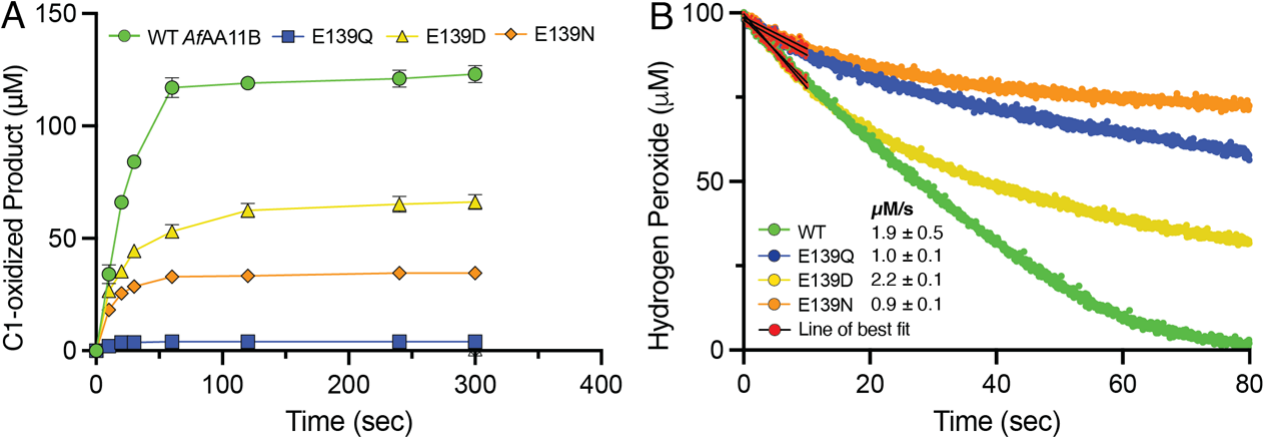
Degradation of (GlcNAc)_4_ by *Af*AA11B variants in reactions with exogeneous hydrogen peroxide. (A) Formation of C1-oxidized product monitored over 300 s and measured using HPAEC-PAD. (B) Hydrogen peroxide consumption monitored over 80 s and measured using a Prussian blue modified rotating gold disc electrode. Both reactions contained 250 nM LPMO, 1 mM ascorbate, 100 μM H_2_O_2_, 1 mM (GlcNAc)_4_, 100 mM KCl, 50 mM Bis–Tris, pH 6.5, and were performed at 37 °C. When monitoring C1-oxidized product, the reaction was incubated in a thermomixer at 750 rpm, while monitoring hydrogen peroxide consumption was performed in an electrochemical chamber with the electrode rotated at an angular velocity of 50 s^–1^. All reported data are the average of 3 independent repeats with the standard deviation reported. For panel B error bars are omitted for clarity.

To gain more information about the initial rate of the reaction, a recently developed hydrogen peroxide sensor for real-time monitoring of hydrogen peroxide consumption in the presence of substrate was used.^37^ Using this method, a measurement can be obtained every 80 ms, enabling an accurate estimation of the initial rate of the reaction prior to enzyme inactivation. WT *Af*AA11B and the E139D mutant showed similar initial rates of activity with 1.9 and 2.2 μM·s^–1^, respectively (at an enzyme concentration of 0.25 μM). In contrast, the E139Q and E139N mutants exhibited approximately half this rate with 1.0 and 0.9 μM·s^–1^, respectively (Figure 6B). None of the three mutants were able to utilize all the supplied hydrogen peroxide before full enzyme inactivation occurred, highlighting the importance of a glutamate at position 139 for productive turnover of hydrogen peroxide and maintaining enzyme integrity. The sensor data confirm that, of the three mutants, E139D is most stable.

As a final assessment of the stability of wild-type and mutant *Af*AA11B, we used the H_2_O_2_ sensor to determine the average number of H_2_O_2_ molecules that the LPMO variants can turn over before being inactivated. This approach was inspired by work of Kuusk et al.,^62^ who studied depletion of ascorbic acid to address the same issue for AA9 and AA10 LPMOs. The results (Figure S11) show that, also in the absence of substrate, the mutants are more prone to damage, showing maximum turnover numbers of 6–15, compared to 37 for the wild-type enzyme. The turnover number obtained for the wild-type enzyme compares well with turnover numbers of 10–40, for AA10 LPMOs, and 100–140, for AA9 LPMOs obtained in the previous study by Kuusk et al.^62^

### A Closer Look at Mutational Effects on the Redox Properties of *Af*AA11B

Fluorescence spectroscopy may be used to assess the redox state of most LPMOs, since the Cu­(I) and Cu­(II) forms of the enzyme give different signals.^63^ The second order rates of reduction by ascorbate and reoxidation by hydrogen peroxide were determined for WT *Af*AA11B and the three Glu139 mutants using stopped-flow fluorescence spectroscopy. The rate of reduction for WT *Af*AA11B was substantially lower compared to all three mutants (Figure 7A), which aligns well with WT *Af*AA11B having the lowest reduction potential (Figure 5B). Looking at the previously reported data for a similar set of variants of *Nc*AA9C^47^ comparable trends emerge. In both studies, the glutamate-containing variants (WT *Af*AA11B and *Nc*AA9C Q164E), exhibited substantially lower rates of reduction than the amine-containing variants (glutamine and asparagine), showing that the impact of this residue on copper reactivity is similar across LPMO families.

**Figure 7:**
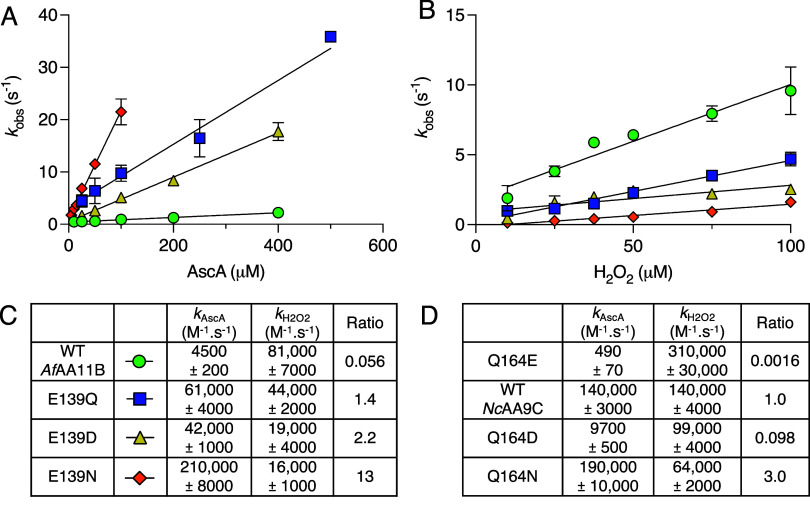
Reduction and reoxidation rates of *Af*AA11B variants determined by fluorescence stopped-flow spectroscopy. (A) Reduction of *Af*AA11B variants by various concentrations of ascorbate (AscA). (B) Reoxidation of *Af*AA11B variants by various concentrations of hydrogen peroxide. For A & B the reactions contained a final concentration of 5 μM LPMO in 50 mM Bis–Tris, pH 6.5. The rate constants (*k*_obs_) were plotted against the various AscA concentrations (A) or H_2_O_2_ concentrations (pseudo-first order) to obtain the apparent second-order rate constants for reduction and oxidation that are reported in C (*k*_AscA_ or *k*_H_2_O_2__, respectively). All experiments were performed at 25 °C. (C) The ratio of the second-order rate constants for reduction and oxidation (*k*_AscA_/*k*_H_2_O_2__) is reported for all *Af*AA11B variants. All reported data are the averages of 3 independent replicates with the standard deviation shown. (D) Previously published reduction (AscA) and reoxidation (H_2_O_2_) rates for the equivalent mutants generated in *Nc*AA9C,^47^ determined in 50 mM Bis–Tris pH 6.5, at 37 °C.

Changes were also observed in the rate of reoxidation by hydrogen peroxide between the *Af*AA11B variants, although these were smaller than the changes in the rate of reduction (Figure 7B). WT *Af*AA11B displayed the highest rate of reoxidation_,_ with the rate of reoxidation being lower for all mutants. The ratios of the rate of reduction and the rate of reoxidation emphasize the large mutational effects on copper reactivity, ranging from 0.056 to 13 (Figure 7C). The variation in these ratios correlates qualitatively, and in an expected manner, with the variation in the redox potential shown in Figure 5B, *i.e.*, the ratio is lower when the redox potential is lower. Again, it is interesting to highlight similarities in copper reactivity effects between *Af*AA11B and *Nc*AA9C.^47^ For both enzymes, the glutamate-containing variants (WT *Af*AA11B and *Nc*AA9C Q164E) exhibited the lowest ratio (0.056 and 0.002, respectively), while the glutamine-containing variants (*Af*AA11B E139Q and WT *Nc*AA9C) both had ratios close to 1 (1.4 and 1.0, respectively).

Of note, the present results represent the first time that rates of reduction and reoxidation have been measured for an AA11 LPMO. The rate of reduction for *Af*AA11B (4500 M^–1^·s^–1^) stands out as an anomaly, being notably lower than the rates reported for other wild-type LPMOs which are typically above (100,000 M^–1^·s^–1^).^42,47^

Despite the similar impact of the glutamate/glutamine exchange on copper reactivity in *Nc*AA9C and *Af*AA11B, the impact of this exchange on enzyme performance during turnover conditions differs strongly between the two enzymes. In both cases, the wild-type enzyme, having a glutamine and a glutamate in *Nc*AA9C and *Af*AA11B, respectively, clearly is most capable of productively using hydrogen peroxide and avoiding damaging off-pathway reactions. To gain more insight into these differences, we investigated the occurrence of hole hopping in *Af*AA11B. Hole hopping is a protective mechanism that involves a series of redox-active amino acids in the core of a protein that redirect potentially damaging holes away from the active site to the protein surface.^64^ This mechanism has been demonstrated in several metalloenzymes,^65,66^ and evidence of hole hopping, or even a complete hole hopping pathway, have been described for AA9 and AA10s LPMOs.^33,35,47,67,68^ Hole hopping has never been shown in LPMOs from the AA11 family and was assessed here using stopped-flow UV–vis spectrophotometry.

A weak UV–visible signal was detected in the 415 nm region for three out of four of the variants tested (Figure 8). This feature is in agreement with what has been previously observed in AA9 LPMOs such as *Nc*AA9C^33^ and *Hj*AA9A.^35^ In these studies, this feature had been assigned to a tyrosyl radical based on EPR and mutagenesis studies. Since the active sites of *Af*AA11B, *Nc*AA9C and *Hj*AA9A all contain a proximally positioned tyrosine residue, it seems safe to predict that the 415 nm feature observed in *Af*AA11B arises from a tyrosyl radical. In contrast to observations made for AA9 and AA10 LPMOs, all *Af*AA11B variants, including the wild-type, lack a feature at about 520 nm, which is predicted to reflect a tryptophanyl radical species. The core of *Af*AA11B contains two tryptophan residues (W129, W131) at 9.4 Å and 5.6 Å from the copper ion, respectively (Figure S12), meaning that these residues are within range for electron transfer.^69,70^ Similarly positioned tryptophan residues occur in *Nc*AA9C (W62, analogous to W129 in *Af*AA11B) and *Hj*AA9A (W79 and W84) (Figure S12) and these are predicted to give rise to the radical species detected at 520 nm.^33,35^ The lack of a 520 nm feature in *Af*AA11B could be due to conformational differences relative to the other two enzymes (Figure S12) that prevent involvement of these tryptophan residues. Alternatively, the formation and decay of a tryptophanyl radical species may be too fast to observe, as it has been previously shown that the formation and decay of this species is much faster compared to the tyrosyl species. In *Hj*AA9A the tryptophanyl species fully decayed after 0.8 s, *versus* 100 s for the tyrosyl species,^35^ and in *Nc*AA9C these rates were 0.07–0.09 and 3–10 s, respectively,^33,47^ demonstrating a much lower lifetime for the tryptophanyl species compared to the tyrosyl species in AA9 LPMOs. The notion that hole hopping through tryptophan residues in *Af*AA11B may be too fast to be observed is supported by the observation that the lifetime of the tyrosyl feature in WT *Af*AA11B (<0.1 s) is orders of magnitude lower than the lifetime (3–100 s) observed in AA9s (Figure 8A–C).

**Figure 8:**
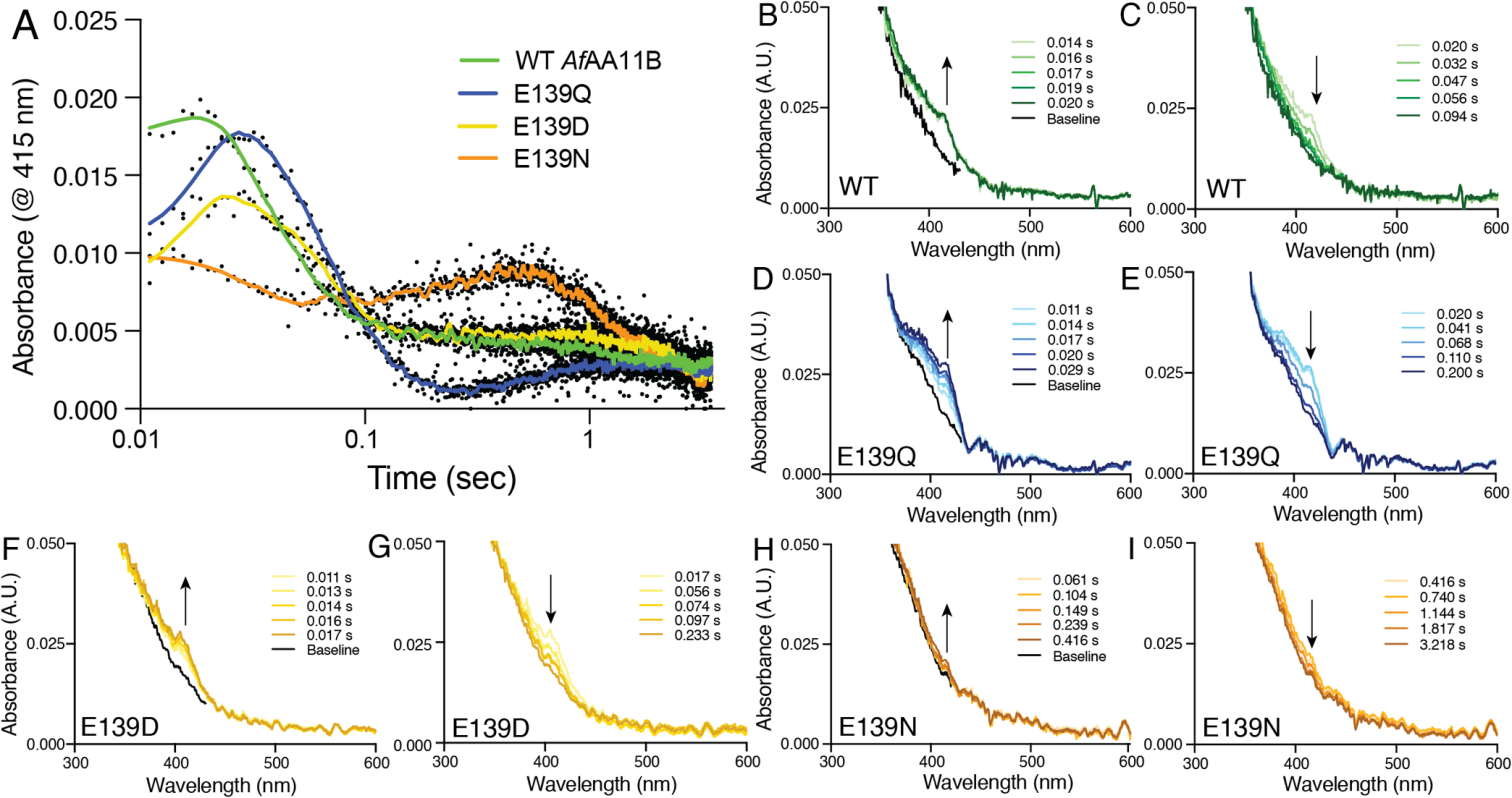
UV–vis stopped flow spectroscopy for *Af*AA11B variants. (A) Formation and decay of the spectral feature at 415 nm. The *X*-axis is shown as a log10 scale to allow better observation of the radical formation phase. A moving data average is shown in the solid-colored lines while the raw data is shown as black dots. (B–I) Radical formation (left) and decay (right), monitored by recording UV–vis absorbance in the 300–600 nm wavelength range over defined time periods. The baseline is shown in black and indicates the theoretical resting state prior to radical formation and is determined from the final decay graphs (*i.e.*, 0.094 s for WT). WT *Af*AA11B (B, C), E139Q (D, E), E139D (F, G) or E139N (H, I) were mixed anaerobically with a one molar equivalent of ascorbate in 50 mM Bis–Tris, pH 6.5, at 4 °C to generate *Af*AA11B–Cu­(I) variants, then mixed with a 20-molar excess of hydrogen peroxide in the absence of substrate. Experiments were acquired in at least duplicate, and all showed comparable results; for clarity only one experiment is shown.

The differences between the different wild-type LPMOs are remarkable and suggest that hole hopping in *Af*AA11B, with its tyrosine-glutamate arrangement, is different, and likely faster compared to *Nc*AA9C and *Hj*AA9A, which both have a tyrosine-glutamine arrangement. This difference is also evident when comparing the mutational effects observed in *Af*AA11B (Figure 8), with those previously observed for *Nc*AA9C. In *Nc*AA9C the Q164E mutation completely abolished the formation and decay of both the tyrosyl and tryptophanyl signals,^47^ while in *Af*AA11B the tyrosyl species was visible and behaved comparably in both WT *Af*AA11B (glutamate-containing) and the E139Q mutant (glutamine-containing) (Figure 8B–E). Differences were also evident in the asparagine-containing variants: In this case, formation and decay of the tyrosyl and tryptophanyl signals in *Nc*AA9C were hardly affected, whereas the glutamate to asparagine mutation made the tyrosyl signal in *Af*AA11B almost invisible (Figure 8H–I). Taken together, these results show that, while the impact of the glutamate/glutamine residue on copper reactivity is similar in the AA9 and the AA11 LPMO (see above), the impacts on enzyme stability in turnover conditions (see Figure 5) and on the efficiency of protective hole hopping reactions (Figure 8) are very different.

## Concluding Remarks

The structural studies described above provide an explanation for the remarkable substrate specificity of *Af*AA11B and, likely, other AA11 LPMOs in the same phylogenetic clade, such as *Ao*AA11. The flexible loop that interacts with chitotetraose is unique for these LPMOs and its discovery provides insight into how LPMOs may have evolved to act on soluble substrates. As pointed out by Rieder et al.,^24^ it does not seem logical that nature has evolved powerful redox enzymes to catalyze a reaction (hydrolysis of soluble oligosaccharides) that can be easily catalyzed by glycoside hydrolases. Perhaps, these LPMOs have evolved to act on other substrates that remain unknown, and just happen to be very active on soluble oligomers. In this respect, it is noteworthy that gene expression studies with *Neurospora crassa* suggest that AA11 LPMOs, which are the most widespread of fungal LPMOs,^22^ play a role in cellular development.^71^

It remains to be seen whether the redox properties of *Af*AA11B, which are unique among wild-type LPMOs, relate to the substrate specificity of this enzyme. Our data show that these redox properties relate directly to the unique tyrosine-glutamate active site arrangement in *Af*AA11B. Interestingly, when it comes to these properties (oxidase activity, redox potential, rates of reduction and oxidation), the various *Af*AA11B variants studied here showed trends similar to those seen in a previous mutational study of *Nc*AA9C, which has a tyrosine-glutamine arrangement.^47^ Thus, when it comes to these properties, the impact of variation in this glutamate/glutamine residue does not seem to depend on other structural or dynamic features of the LPMOs. Differences in such features include the presence of a third His in *Nc*AA9C (at 4.9 Å from the active site copper and 3.6 Å from the glutamine), which may play a role in hydrogen peroxide stabilization alongside the glutamate/glutamine^36,43,44^ and which is absent in *Af*AA11B (Figure 4A,D). It should be noted that the apparent correlations between certain structural features and the oxidase activity, as well as the apparent correlation between a lower redox potential and a higher oxidase activity, require further studies. The oxidase reaction in LPMOs is complex and not fully resolved, and may vary between LPMO types.^45,48,72–74^ For example, while it intuitively seems logical that a lower reduction potential and faster oxidation of the copper, correlate with higher oxidase activity, the initial reaction of reduced copper with molecular oxygen may not be rate-limiting in all conditions and for all LPMOs.

The analysis of peroxygenase reactions and protective hole hopping mechanisms confirmed that Glu139 is of crucial importance to ensure productive use of hydrogen peroxide and somehow affects hole hopping. Previous studies of *Ls*AA9A,^43^ *Nc*AA9C,^47^ *Ta*AA9A^75^ and *Sm*AA10A^42^ have shown that the glutamine or glutamate at this position plays a role in positioning the hydrogen peroxide and confining emerging intermediates. Importantly, the present results show that the impact of a glutamate, glutamine, aspartate or asparagine at the Glu139 position on the productive LPMO reaction is strongly LPMO-context dependent. Of the four variants tested, WT *Af*AA11B, with a glutamate, was clearly the most efficient peroxygenase. Likewise, of the four similar *Nc*AA9C variants tested,^47^ the wild-type was again the best enzyme, and this wild-type contains a glutamine. Clearly, other features that vary between the two LPMOs play a role. These could be the features mentioned above (third His, dynamics) or other features that have not yet been recognized as being important.

Our analysis of hole hopping in *Af*AA11B, which is the first study of this sort for AA11 LPMOs, revealed huge differences between LPMO families and suggests that hole hopping in *Af*AA11B is exceptionally fast. The impact of the various mutations further emphasizes the differences between LPMOs as the mutational effects observed here differ considerably from those previously observed for *Nc*AA9C. Variation in hole hopping routes and efficiencies could reflect evolutionary adaptations to the specific function of the LPMO and to the (redox) environment it has evolved to act in. Hole hopping mechanisms have likely evolved as an escape route for reactive holes and will prolong enzymatic activity, possibly at the expense of substrate oxidation.^68^ It is therefore imperative that the balance between productive catalysis and the protective hole hopping mechanism is fine-tuned to create an efficient LPMO. *Af*AA11B, acting on soluble substrates, is a fast peroxygenase and, therefore, perhaps also needs a fast hole hopping mechanism to protect the enzyme. Further studies aiming at finding causal relationships between the variation in hole hopping mechanisms and efficiency on the one hand, and LPMO performance on the other hand, would be of major interest.

In conclusion, this study provides a structural explanation for the unique substrate specificity of *Af*AA11B, sheds light on features underlying the remarkable redox properties of this enzyme and reveals the context-dependent impact of the conserved glutamate/glutamine residue on the peroxygenase activity and turnover stability of LPMOs. While this study highlights certain parallels between AA9 and AA11 LPMOs, it emphasizes distinctive differences between the two families. This shows that a one-size-fits-all approach is not suitable when studying LPMO catalysis, as each family has its own set of unique features. The findings presented in this study contribute to our understanding of catalysis in the AA11 LPMO family, which is the most widespread family of fungal LPMOs.

## Materials and Methods

### Chemicals and Reagents

All chemicals used were ordered from Carl Roth (Karlsruhe, Germany), VWR (Radnor, PA) or Sigma-Aldrich (St. Louis, MO). Oligonucleotides were ordered from Integrated DNA Technologies (Leuven, Belgium). For plasmid isolation (Wizard Plus SV Minipreps DNA Purification Systems) and purification of fragments resulting from PCR or restriction enzyme digests (Wizard SV Gel and PCR Clean-Up System), kits were purchased from Promega (Fitchburg, WI). For cloning by Gibson isothermal assembly, the method described by Gibson et al.,^76^ was used. All other enzymes and Phusion DNA polymerase were obtained from Thermo Fisher Scientific (Waltham, MA).

### Cloning

The pBSY3Z-P_*DAS2*_ Escherichia coli/K. phaffii shuttle vector, a variant of the commercially available pBSY3Z plasmid (bisy GmbH, Hofstätten an der Raab, Austria), was used to produce WT *Af*AA11B and the mutants thereof. Besides the regulatory elements required for plasmid propagation and maintenance in E. coli during the cloning procedure, the pBSY3Z plasmid contains the PDC promoter for transcription initiation and the *AOX1* terminator (*AOX1*TT) for termination of the transcription of the gene of interest. To ensure tight and inducible transcriptional regulation, the derepressed PDC promoter was exchanged with the strong methanol inducible *DAS2* promoter (P*_DAS2_* ^72^). For this, the pBSY3Z backbone^77^ was PCR amplified using primer pair one (Table S1) and the P*_DAS2_* was amplified from K. phaffii genomic DNA using primer pair two (Table S1). The primers were designed so that the fragment containing P*_DAS2_* has 30 bp regions homologous to the pBSY3Z backbone, as required for Gibson assembly. For construct assembly, the two PCR fragments were mixed in a 1:3 molar ratio (pBSY3Z:P*_DAS2_*) and incubated at 50 °C for 60 min in the presence of the Gibson assembly mixture. Next, 2.5 μL of the assembly mixture was used to transform in-house generated chemically competent E. coli XL1-Blue cells (Mix & Go! E. coli Transformation Kit and Buffer Set, Zymo Research, Irvine, CA). The recovered cells were plated on LB (Luria/Miller) agar plates containing 25 μg·mL^–1^ Zeocin. Positive transformants were cultivated in/on LB-medium containing 25 μg·mL^–1^ Zeocin for plasmid propagation and plasmids were isolated using the appropriate kit. The resulting plasmid (pBSY3Z-P*_DAS2_*) was sequence verified by Sanger sequencing (Microsynth AG, Balgach, Switzerland).

To clone in the *Af*AA11B gene, the stuffer fragment separating the P*_DAS2_* and *AOX1*TT regions in the pBSY3Z-P*_DAS2_* vector was removed by PCR amplification using primer pair three (Table S1). The K. phaffii codon-optimized gene encoding the WT *Af*AA11B (NCBI accession number XP_748042.1) was amplified from a previously made expression plasmid,^24^ including the Ost1 signal peptide instead of the native signal peptide^78^ using primer pair four (Table S1). This also introduced 30 bp regions homologous to the promoter and terminator of the expression cassette on the pBSY3Z-P*_DAS2_* vector. The fragments were assembled and used to transform E. coli as described above. To generate the *Af*AA11B E139D, E139N, E139Q and N99A mutants, pBSY3Z-P*_DAS2_*-*Af*AA11B-WT was amplified using primer pairs five, six, seven and eight, respectively (Table S1). To generate the truncated version of WT *Af*AA11B, *i.e.*, without the linker and the X278 module (residues S220-A397), a PCR using primer pair nine (Table S1) was performed, removing these residues. These primers (sets 5–9) were designed to have complementary overhangs to allow circularization of the PCR products when incubated with the Gibson assembly mixture. Transformation of E. coli and plasmid propagation and isolation were done as described above. All expression plasmids were sequence verified prior to use.

### K. phaffii Transformation

As the production host, we used the killer plasmid-free K. phaffii strain BSYBG11 (Δ*AOX1*, Mut^S^). Preparation of electrocompetent K. phaffii cells and transformations were done as described by Lin-Cereghino et al.,^79^ with minor deviations. In brief, 2000 ng of the circular plasmids were linearized using *Smi*I, according to the manufacturer’s directions, followed by desalting using 13 mm 0.025 μm MCE membranes (Merck Millipore Ltd., Cork, Ireland). For K. phaffii transformation, 80 μL of the competent K. phaffii cells were mixed with 1000 ng of the linearized and desalted DNA in an electroporation cuvette (2.0 mm gap) and incubated for 5 min on ice. Subsequently, the cell-DNA mixture was pulsed using an electroporator with the following settings: 2 kV; resistance 200 Ω; capacitance, 25 μF. Then 500 μL of a 1:1 mixture of YPD (1% (w/v) yeast extract, 2% (w/v) peptone, 2% (w/v) glucose) and 1 M sorbitol was added to the cells followed by incubation at 28 °C, 250 rpm for 1.5 h. The recovered cells were then plated on YPD plates supplemented with 100 μg·mL^–1^ Zeocin and incubated for two to 3 days at 28 °C until colonies were visible.

### Identification of High Producing K. phaffii Strains

Ectopic genomic integration of expression constructs results in K. phaffii transformants with different expression levels (clonal variation). To identify clones with high expression levels, the amount of secreted LPMO was assessed for 72 transformants of each construct. For this, microscale cultivation was done as described by Weis et al.,^80^ with LPMO secretion levels assessed as described previously.^24^ In brief, 72 transformants were randomly selected and used to inoculate 250 μL buffered minimal medium (BM; 200 mM potassium phosphate, pH 6.0, 1.34% (w/v) yeast nitrogen base (YNB), 4 × 10^–5^ % (w/v) biotin) supplemented with 1% (w/v) dextrose in a 96-well deep-well plate. After 60 h of cultivation at 28 °C and 320 rpm, LPMO production was induced by adding 250 μL BM medium supplemented with 1% (w/v) methanol to each well. Over the remaining cultivation time, protein production was induced three times with regular intervals of approximately 24 h by adding 50 μL BM medium supplemented with 5% (w/v) methanol. After ∼96 h of total cultivation, the cells were separated from the supernatant by centrifugation at 5000*g* at 4 °C for 10 min. The secretion level of the recombinant LPMOs was assessed using the Bradford assay and was confirmed by SDS-PAGE analysis. The best-secreting K. phaffii strains for each construct were selected for further use.

### Production and Harvesting (Shake Flasks)

Full-length *Af*AA11B variants were produced as follows. Briefly, 2 L baffled shake flasks containing 450 mL of BM medium supplemented with 1% (w/v) dextrose were inoculated with a single colony and incubated at 28 °C, 180 rpm for approximately 66 h. After 66 h, expression was induced with the addition of 50 mL of BM medium supplemented with 5% (w/v) methanol, followed by incubation for a further 8 h. Following this, 5 mL of 100% pure methanol was added every 12 h to maintain induction. After 120 h total incubation, cells were harvested by centrifugation, and the protein-containing supernatant was filtered through a 0.22 μm Steritop filter (Merck Millipore, Burlington, MA). The supernatant was then concentrated 10-fold using a VivaFlow 200 tangential flow filtration system with a molecular weight cutoff (MWCO) of 10 kDa (Sartorius, Göttingen, Germany).

### Production and Harvesting (Bioreactor)

Truncated WT *Af*AA11B was produced in a fed-batch cultivation process using a 6.9 L Biostat Cplus bioreactor (Sartorius Stedim, Göttingen, Germany). Media for the batch and fed-batch phase was prepared according to Gasset et al.,^81^ using glycerol instead of glucose as the carbon source. The bioreactor was set to 28 °C and pH 5.0 which was controlled using 15% (v/v) NH_4_OH. For the batch phase, 3 L of the bioreactor batch medium containing 4% (w/v) glycerol were inoculated to an OD_600_ of 1 using ∼200 mL of an overnight culture. For the overnight culture, a single yeast colony was used to inoculate 400 mL YPG [1% (w/v) yeast extract, 2% (w/v) peptone, 1% (w/v) glycerol] in a 2.5 l Thomson’s Ultra Yield Flask. Once the batch phase was completed (as indicated by increasing oxygen levels in the bioreactor and decreased need for NH_4_OH), an exponential feed was started using a fed-batch medium with 40% (w/v) glycerol to keep the growth rate stable. After a biomass concentration of ∼35 g·L^–1^ dry cell weight was reached, the production of truncated WT *Af*AA11B was induced by injecting 2 g·L^–1^ of pure methanol every 90 min over the final 14 h of cultivation. The accumulation of protein in the culture supernatant was monitored using the Bradford assay and SDS-PAGE analysis. Cells were harvested by centrifugation and the protein-containing supernatant was filtered through a 0.22 μm Steritop filter (Merck Millipore, Burlington, MA). The supernatant was then concentrated 10-fold using a Centramate 500 S Tangential Flow Filtration (TFF) system (Pall, East Hills, NY) equipped with a membrane with a 10 kDa MWCO.

### Purification and Copper Saturation

Both the full-length *Af*AA11B variants and truncated WT *Af*AA11B were purified using two purification steps: hydrophobic interaction chromatography (HIC) and size exclusion chromatography (SEC). A 5 mL HiTrap Phenyl FF column (Cytiva, Marlborough, MA) was equilibrated with 50 mM Bis–Tris pH 6.5, 2.4 M ammonium sulfate. Ammonium sulfate was added to the concentrated supernatant to a final concentration of 2.4 M and loaded onto the equilibrated column. The protein was eluted by applying a linear gradient of 0–100% 50 mM Bis–Tris pH 6.5 over 40 mL at a flow rate of 2 mL·min^–1^. Protein purity was assessed using SDS-PAGE and fractions containing protein of the correct size were pooled together and concentrated to 1 mL using an Amicon Ultra-15 3 kDa MWCO centrifugal filter unit (Merck Millipore, Burlington, MA). As the protein was relatively pure prior to SEC, copper saturation was performed on pooled HIC fractions and SEC was used to simultaneously remove excess free copper and remaining contaminating proteins present in the sample. A 3-fold molar excess of Cu­(II)­SO_4_ was added to the concentrated protein sample followed by incubation on ice for 60 min. A HiLoad 16/600 Superdex 75 size exclusion column (Cytiva, Marlborough, MA) was equilibrated with 50 mM Bis–Tris pH 6.5, 200 mM NaCl, and the copper saturated protein sample (1 mL) was loaded onto the column using a flow rate of 1 mL·min^–1^. The protein started to elute after approximately 50 min for full-length *Af*AA11B variants and after approximately 68 min for truncated WT *Af*AA11B. Protein purity was assessed using SDS-PAGE and fractions containing pure protein were concentrated and buffer exchanged into 50 mM Bis–Tris pH 6.5 using an Amicon Ultra-15 3 kDa MWCO centrifugal filter unit (Merck Millipore, Burlington, MA). Proteins were stored at 4 °C.

### Crystallography

Crystallization trials were performed with truncated WT *Af*AA11B that had been de-*N*-glycosylated using an endo-β-*N*-acetylglucosaminidase from Enterococcus faecalis (*Ef*Endo18A), which was produced in-house according to Bøhle et al.,^82^ 1 μM of *Ef*Endo18A was mixed with 5 mg of truncated WT *Af*AA11B followed by incubation for 30 min at room temperature. *Ef*Endo18A contains a His-tag and was subsequently removed by adding 1 mL Ni-NTA resin (Qiagen, Hilden, Germany) to the reaction mixture. Unbound de-N-glycosylated truncated WT *Af*AA11B was eluted from the column using 50 mM Bis–Tris, pH 6.5, and was concentrated to 15 mg·mL^–1^ using an Amicon Ultra-15 3 kDa MWCO centrifugal filter unit (Merck Millipore, Burlington, MA). Crystal screens were set up using the Hampton Research Index kit (Aliso Viejo, CA). The reservoir solution was placed in a 48-well VDX plate (Hampton Research, Aliso Viejo, CA) and hanging drops were created by mixing equal volumes of 15 mg·mL^–1^ deglycosylated truncated WT *Af*AA11B and reservoir solution to a final volume of 2 μL. The wells were sealed using 12 mm siliconized glass cover slides (Hampton Research, Aliso Viejo, CA), and stored in the dark at room temperature. Crystals suitable for X-ray crystallography were obtained in one condition (0.1 M Tris pH 8.5, 2 M ammonium sulfate) after approximately 3 months (Figure S3). Crystals deemed suitable for X-ray crystallography were transferred to a solution consisting of reservoir solution supplemented with 10 mM ascorbic acid and incubated for 10 min, followed by a cryosolution containing the reservoir solution supplemented with 22% (w/v) glucose prior to flash-freezing in liquid nitrogen.

### Data Collection and Structure Determination

Diffraction data were collected at the ID30A-3 beamline (Dectris Eiger X 4 M detector) at the European Synchrotron Radiation Facility (ESRF, Grenoble, France). The data was processed using the EDNA pipeline^83^ at ESRF, including programs for indexing and integrating data (XDS^84^), and scaling and merging integrated intensities (AIMLESS^85^). The data were truncated from 1.32 to 1.40 Å using the program TRUNCATE^86^ within the CCP4 ^87^ package. The structure was solved by the molecular replacement program Phaser^88^ from the Phenix^89^ program package, using a homology model based on PDB: 4MAH as a search model. Refinement was performed using PHENIX.refine^90^ and model manipulations were performed in *Coot*.^91^ Alternate cycles of positional refinement in Phenix and manual rebuilding in *Coot* were carried out until all residues possessed well-defined electron density and no further improvements of the *R*_work_ and *R*_free_ factors were observed. Data collection and refinement statistics are summarized in Table 1. Structure factors and coordinates have been deposited in the Protein Data Bank (PDB accession number: 9HDG).

### Loop Prediction and Molecular Dynamics Simulations

The missing loop in the crystal structure (residues 152–172) was reconstructed using different independent methods: AlphaFold,^55^ RoseTTAFold,^56^ SwissModel^57^ and RosettaRemodel.^58^ Molecular dynamics simulations were performed on the best model from each of the four prediction methods to evaluate the stability of the reconstructed loop. The protonation states of ionizable amino acids at pH 7.0 were assigned using the PROPKA software.^92^ The models were solvated with a 10 Å layer of TIP3P water and Na^+^/Cl^–^ ions to neutralize the system charge, resulting in a final simulation box of ≈30.000 atoms. The proteins were modeled without copper ions and geometric constraints were applied to retain the histidine brace geometry, which is a known, highly conserved structural feature of LPMOs.^93^ Specifically, a harmonic constraint was used to keep the crystallographic distances between the three nitrogen atoms of the histidine brace, using a 50 kcal·mol^–1^·Å^–2^ spring constant. The models were subjected to 5000 steps of energy minimization with 5 kcal·mol^–1^·Å^–2^ positional restraints on the backbone atoms of the enzyme. The systems were then heated linearly from 0 to 300 K for 100 ps at a constant volume, with restraints lowered to 1 kcal·mol^–1^·Å^–2^, using the Langevin thermostat with a collision frequency of 1 ps^–1^. Density equilibrations were performed at 300 K for 1 ns at a constant pressure of 1 atm using the Berendsen barostat with a pressure relaxation time of 1 ps. The final 50 ns equilibration step was performed in the NVT ensemble. During this equilibration stage, all positional restraints were removed. The 1 μs production runs were performed using the same conditions as in the final equilibration step. In all simulations, we used a time step of 2 fs, periodic boundary conditions with a 12 Å cutoff for nonbonded interactions, and PME treatment of long-range electrostatics. Simulations were performed using the CUDA version of NAMD3 ^94^ and the CHARMM 36 force field.^95^ Preprocessing and postprocessing of the MD simulations were performed using VMD^96^ and PyMOL.

### Chitin Complex Molecular Dynamics Simulations

*Af*AA11B and *Af*AA11A (PDB7P3U) were simulated in complex with crystalline β-chitin and chitin oligomers (dimer, tetramer and hexamer). The histidine brace including a Cu­(I) ion was modeled used parameters developed in a previous study.^97^ The input structures for the generated complexes with crystalline chitin were based on a previous model,^42,97^ by aligning the histidine brace of *Af*AA11A or *Af*AA11B with the histidine brace of *Sm*AA10A present in that model. Models of *Af*AA11A and *Af*AA11B in complex with chitohexaose, chitotetraose or chitobiose were generated by trimming the crystalline chitin, leaving only part of the single chitin chain that is binding to the catalytic copper site (see Figure S7). Tetramers were modeled by considering two different placements, binding from subsite −3 to +1 and from subsite −2 to +2. MD simulations were carried out as described above, restraining the position of C1 atoms of all chitin monomers in the equilibration stages. The production MD simulations of *Af*AA11B and *Af*AA11A in complex with crystalline β-chitin were run for 100 ns, and during the simulations 1 kcal·mol^–1^·Å^–2^ positional restraints were applied to the C1 atoms of the lowest layer of chains of the crystalline chitin model (the “highest” layer being the one interacting with the LPMO). The production MD simulations of *Af*AA11B and *Af*AA11A in complex with chitin oligomers were run for 200 ns, with no positional restraints.

### Activity Assays with Chitotetraose (GlcNAc)_4_

Reactions performed under apparent monooxygenase conditions, *i.e.*, reactions driven by reductant oxidation, contained 1 μM LPMO and 1 mM (GlcNAc)_4_ (Megazyme, Bray, Ireland) in 50 mM Bis–Tris, pH 6.5, and were initiated by adding 1 mM ascorbate. Reactions were incubated at 37 °C, 750 rpm, in an Eppendorf Thermomixer with samples taken over a 240 min period. Reactions containing exogenous hydrogen peroxide were performed in a similar manner and contained 250 nM LPMO, 100 μM hydrogen peroxide and 1 mM (GlcNAc)_4_ in 50 mM Bis–Tris, pH 6.5, and were initiated by adding 1 mM ascorbate. Reactions were incubated at 37 °C, 750 rpm in an Eppendorf Thermomixer with 50 μL samples taken over a 300-s period. All reactions were stopped by adding the 50 μL reaction samples to 150 μL of 200 mM NaOH followed by filtering through a 0.45 μm filter plate. The resulting filtrates were stored at −20 °C prior to analysis. The concentration of hydrogen peroxide in stock solutions was determined by measuring the absorbance at 240 nm and using the extinction coefficient of 43.6 M^–1^·cm^–1^. All reactions were performed under aerobic conditions.

### Analysis of C1-Oxidized Products

C1-oxidized dimer and trimer products generated from the degradation of (GlcNAc)_4_ by *Af*AA11B, were detected and quantified by high performance anion exchange chromatography with pulsed amperometric detection (HPAEC-PAD) as described previously.^24^ HPAEC-PAD was performed using a Dionex ICS5000 system equipped with a CarboPac PA200 analytical column and a CarboPac PA200 guard column. Product separation was achieved by applying an 18 min stepwise gradient with varying amounts of eluent A and B (eluent A: 0.1 M NaOH; eluent B: 1 M sodium acetate, 0.1 M NaOH), using a flow rate of 0.5 mL·min^–1^. The gradient consisted of 0–10% eluent B over 5 min, 10–100% eluent B over 3.5 min, 100% eluent B for 1 min, 100–0% eluent B over 0.1 min and finally 0% eluent B for the remaining 8.5 min. C1-oxidized products were quantified using in-house produced C1-oxidized standards generated by reacting chitooligosaccharides (DP2-DP3) with chitooligosaccharide oxidase (ChitO).^98,99^ Chromatograms were recorded and analyzed using Chromeleon 7.

### Real-Time Monitoring of Hydrogen Peroxide Consumption

Hydrogen peroxide-driven LPMO activity in the presence or absence of substrate was monitored by fast electrochemical detection of the depletion of hydrogen peroxide, as originally described by Schwaiger et al.,^37^ and following the procedure detailed in Ayuso-Fernández et al.^68^ The conditions used varied slightly, as indicated for each of the reported experiments.

### Hydrogen Peroxide Production Assay

Hydrogen peroxide production (oxidase activity) was measured using a modified Amplex Red and horse radish peroxidase (HRP) assay as described previously^39,100^ and was performed in the absence of substrate. Amplex Red Reagent (Thermo Fisher Scientific, Waltham, MA) was dissolved in DMSO to 10 mM and HRP (Sigma-Aldrich, St. Louis, MO) was dissolved in 50 mM Bis–Tris pH 6.5 to 100 U.mL^–1^. Reactions contained 1 μM LPMO, 5 U·mL^–1^ HRP and 100 μM Amplex Red Reagent in 50 mM Bis–Tris, pH 6.5 and were incubated at 30 °C for 5 min. The reaction was initiated by adding ascorbate to a final concentration of 1 mM and incubated at 30 °C for 40 min, while monitoring the formation of resorufin by following the absorbance at 540 nm in a Multiskan FC microplate photometer (Thermo Fisher Scientific, Waltham, MA). A hydrogen peroxide standard curve was prepared in the same manner with ascorbate added prior to the addition of Amplex Red and HRP.

### Reduction Potential

The reduction potential of the *Af*AA11B variants was measured using reduced TMP (*N*,*N*,*N*′,*N*′-tetramethyl-1,4-phenylenediamine) as described previously.^51^ TMP_red_ and LPMO-Cu­(II) at final concentrations of 150 μM and 35 μM, respectively, in 20 mM Pipes buffer, pH 6.0, were incubated at 25 °C and left to reach equilibrium in anaerobic conditions (approximately 5 min). The equilibrium constant was determined by measuring the absorbance of TMP_ox_ at 610 nm. The equilibrium constant was used to measure the cell potential of the LPMO-Cu­(II)/LPMO-Cu­(I) redox couple. The reported values are shifted to be referenced against a normal hydrogen electrode (NHE).

### Fluorescence Stopped-Flow Spectroscopy

The rate of reduction and reoxidation of the LPMO variants was measured using the intrinsic differences in fluorescence between the Cu­(II) and Cu­(I) states of the LPMO. All experiments were performed with a SFM4000 stopped-flow rapid spectrophotometer (BioLogic, Seyssient-Pariset, France) coupled to a photomultiplier with an applied voltage of 600 V for detection. The excitation wavelength was set to 280 nm, and the fluorescence increase (for reduction) or decay (for reoxidation) was monitored using a 340 nm bandpass filter. The observation head was equipped with a FC-15/7.5 cuvette to maximize the fluorescence detected at a 90° angle. Single mixing experiments were performed to measure the reduction of LPMO-Cu­(II) to LPMO-Cu­(I) by ascorbate. A 10 μM LPMO-Cu­(II) stock solution (5 μM final concentration) was mixed with equal volumes of ascorbate solutions with varying concentrations (10–500 μM final) and the increase in fluorescence was detected. Double mixing experiments were performed to measure the reoxidation of LPMO-Cu­(I) to LPMO-Cu­(II) by hydrogen peroxide. In this setup a 20 μM stock solution of LPMO-Cu­(II) (5 μM final concentration) was mixed with one molar equivalent of l-cysteine and incubated for 15 s to form LPMO-Cu­(I). Subsequently, *in situ* generated LPMO-Cu­(I) was mixed with solutions containing varying concentrations of hydrogen peroxide (10–100 μM final) and the fluorescence decay was measured. All experiments were performed at 25 °C in 50 mM Bis–Tris, pH 6.5, and were performed in at least triplicates. All reagents were deoxygenated using a Schlenk line with N_2_ flux and prepared in sealed syringes in an anaerobic chamber. The stopped-flow rapid spectrophotometer was flushed with a large excess of anaerobic buffer before attaching the sealed syringes and performing the experiments.

### Kinetics Data Analysis

Fluorescence data collected with the stopped-flow method was analyzed by fitting it to a single exponential function (y = *a*t + *b* + *c*·e^–*k*_obs_·*t*^). This analysis was performed using the BioKine32 V4.74.2 software (BioLogic, Seyssient-Pariset, France) to obtain the first-order rate constant (*k*_obs_) for each concentration of ascorbate or hydrogen peroxide. Subsequently, the obtained rate constants, *k*1_obs_ for ascorbate and *k*2_obs_ for hydrogen peroxide, were plotted against their respective concentrations. These plots were fitted by linear least-squares regression using GraphPad Prism 9 to calculate the apparent second-order rate constants for the reduction step (*k*1_app_^ascorbate^) and reoxidation step (*k*2_app_^H_2_O_2_^).

### UV–Vis Stopped-Flow Spectroscopy

The formation and decay of UV–visible features were measured using UV–vis stopped-flow spectroscopy in double mixing experiments. In this case, a TC-100/10 cuvette with a 1 cm path length was installed in the observation head and a TIDAS S 500 MCS UV/NIR 1910 diode array (J&M Analytik AG, Essingen, Germany) was used as detector. A stock solution of 350 μM LPMO-Cu­(II) (87.5 μM final concentration) was mixed with one molar equivalent of ascorbate and incubated for 10 s to generate LPMO-Cu­(I). Subsequently, LPMO-Cu­(I) was mixed with a 20-molar equivalent of hydrogen peroxide and the formation and decay of UV–visible features was monitored. All experiments were performed at 4 °C in 50 mM Bis–Tris, pH 6.5, and were performed in at least duplicates. A Fisher Scientific Polystat 36 Chiller (Waltham, MA) was used to ensure a reaction temperature of 4 °C during analysis. All reagents were deoxygenated using a Schlenk line with N_2_ flux and prepared in sealed syringes in an anaerobic chamber. The stopped-flow rapid spectrophotometer was flushed with a large excess of anaerobic buffer before attaching the sealed syringes and performing the experiments.

## Supplementary Material



## Notes

Full phylogenetic tree of AA11 LPMO catalytic domains; supporting data for the crystallography; additional *in silico* analysis of AA11 binding surfaces and *Af*AA11B loop configurations; models and results for molecular dynamics simulations; characterization of the N99A mutant; analysis of ascorbate peroxidase activity; and a list of primers (PDF)
