# Supplementary material for: Structure–Function Analysis of an Understudied Type of LPMO with Unique Redox Properties and Substrate Specificity
Source: ACS Catal. 2025 Jun 6;15(12):10601–17. doi: 10.1021/acscatal.5c03003 (PMC12186267; doi:10.1021/acscatal.5c03003)
Supplement: Supplementary file 1 [file cs5c03003_si_001.pdf]

Supporting Information for

Structure-function analysis of an understudied type of LPMO with unique redox properties and substrate specificity

Kelsi R. Hall[1,4], Synnøve Elisa Rønnekleiv[1], Alfonso Gautieri[2], Hedda Lilleås[1], Rannei Skaali[1], Lukas Rieder[1,3], Andrea Nikoline Englund[1], Eirin Landsem[1], Tom Z. Emrich-Mills[1], Iván Ayuso-Fernández[1], Åsmund Kjendseth Røhr[1], Morten Sørlie[1] & Vincent G. H. Eijsink[1*]

[1] Faculty of Chemistry, Biotechnology and Food Science, Norwegian University of Life Sciences (NMBU), 1432, Ås, Norway.

[2] Biomolecular Engineering Lab, Dipartimento di Elettronica, Informazione e Bioingegneria, Politecnico di Milano, Piazza Leonardo da Vinci 32, 20133 Milano, Italy.

[3] Institute for Molecular Biotechnology, Graz University of Technology, 8010, Graz, Austria.

[4] Biomolecular Interaction Centre and School of Biological Sciences, University of Canterbury, PO Box 4800, Christchurch 8140, New Zealand.

* Correspondence to: vincent.eijsink@nmbu.no

This PDF file includes:

1. List of Supplementary Figures & Tables pg. 2
2. Supplementary Figures (1-12) and Table (1) pg. 3-15
3. Supplementary References pg. 16

1. List of Supplementary Figures and Tables

Figure S1. Maximum likelihood tree of the catalytic domains of AA11 LPMOs.

Figure S2. Purified *Af*AA11B used for crystallography screens.

Figure S3. Crystals of the catalytic domain of wild-type *Af*AA11B formed by hanging drop diffusion.

Figure S4. Binding surface of AA9, AA10 and AA11 LPMOs.

Figure S5. Dendrogram showing structural clustering of LPMO structures.

Figure S6. Molecular models of *Af*AA11B with reconstructed loop 152-172.

Figure S7. Initial molecular models of *Af*AA11A in complex with crystalline β-chitin and chitin oligomers.

Figure S8. Molecular dynamics simulation of *Af*AA11B in complex with crystalline chitin.

Figure S9. Distance between chitin oligomers and the histidine brace of *Af*AA11A and *Af*AA11B as observed during 250 ns MD simulations.

Figure S10. Activity of the N99A mutant of *Af*AA11B on chitotetraose.

Figure S11. Impact of mutating Glu139 on inactivation of *Af*AA11B in the ascorbate peroxidase reaction.

Figure S12. Structural alignment of *Af*AA11B, *Nc*AA9C and *Hj*AA9A.

Table S1. List of primers used for cloning the *Af*AA11B variants.

2. Supplementary Figures and Tables

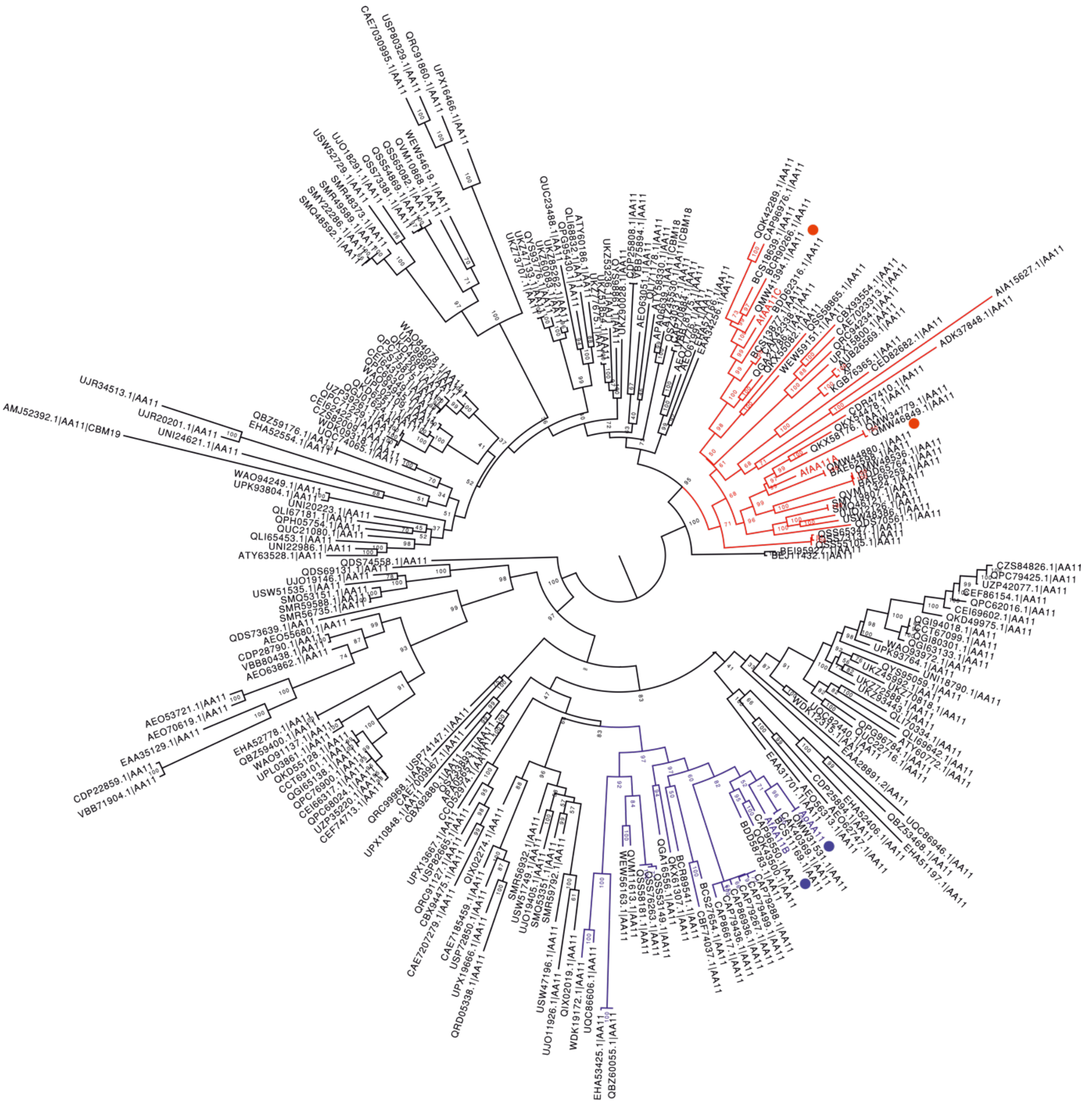

Figure S1. Maximum likelihood tree of the catalytic domains of AA11 LPMOs. The 238 amino acid sequences used in this study were obtained from dbCAN and curated as described in the Materials and Methods. The subclades containing the LPMOs discussed in this study are highlighted in red (*Af*AA11C and *Af*AA11A) or blue (*Ao*AA11 and *Af*AA11B). The four specific LPMOs are labelled with red and blue bullets, respectively. The sequences were aligned using MAFFT and the tree was obtained with IQ-Tree. Bootstrap values of 1000 replicates are indicated at the nodes.

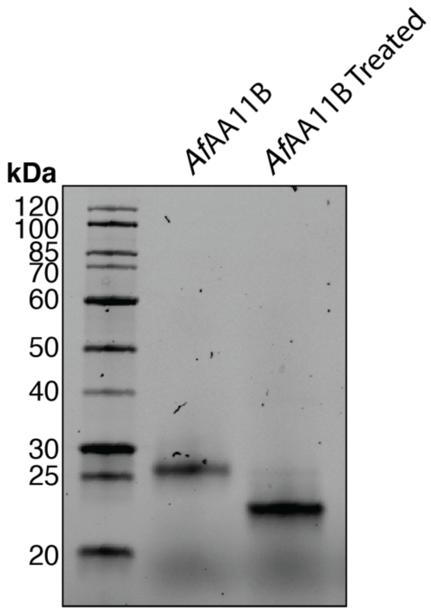

Figure S2. Purified *Af*AA11B used for crystallography screens. Mature *Af*AA11B has an estimated size of 23.4 kDa, however *O*- and/or *N*-linked glycosylation by *K. phaffii* makes the protein larger than this, as shown in lane 2. Lane 3 shows the protein following treatment with *Ef*Endo18A that cleaves off *N*-linked glycosyl groups, resulting in a band closer to the estimated 23.4 kDa size. The treated protein was used in crystallography screens. Lane 1 contains a protein ladder, with protein weights indicated in kDa.

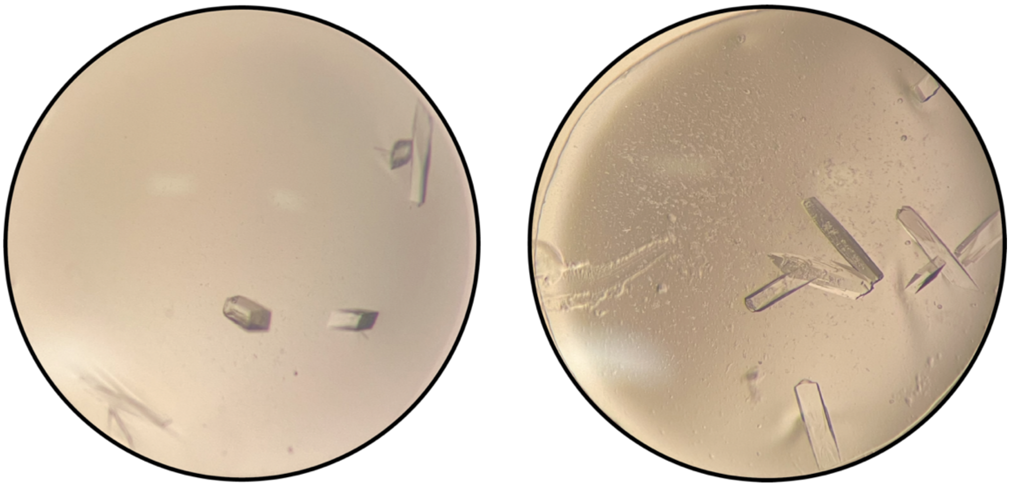

Figure S3. Crystals of the catalytic domain of wild-type *Af*AA11B formed by hanging drop diffusion. Crystals were formed in conditions containing 0.1 M Tris, pH 8.5, and 2 M ammonium sulphate in 48-well VDX plates. The plates were stored in the dark at room temperature and crystals were obtained after approximately 5 months. These crystals were used for collecting diffraction data.

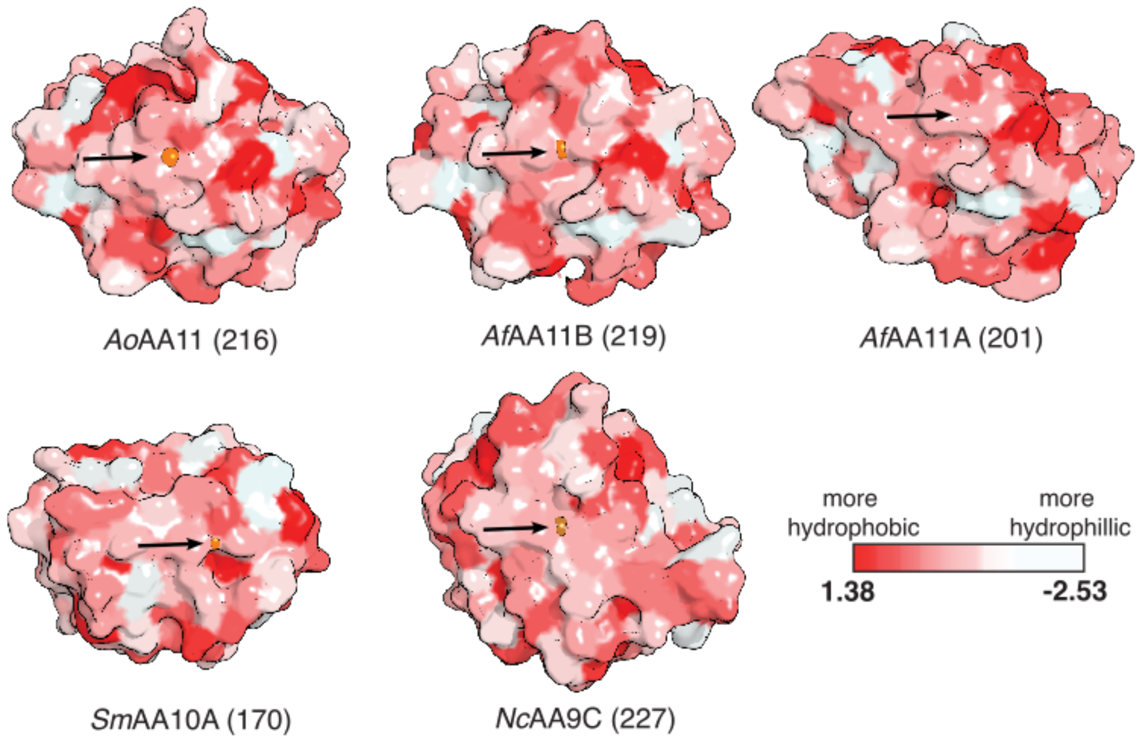

Figure S4. Binding surface of AA9, AA10 and AA11 LPMOs. The flat binding surface of each LPMO is shown in the same orientation, facing towards the reader. The copper ion (orange sphere) is partially hidden beneath the surface and its position is indicated by black arrows. *Af*AA11A does not have a modelled copper therefore the predicted location of the copper is denoted. The surfaces are coloured in accordance with the Eisenberg hydrophobicity scale[1]. The names of the LPMOs are followed by the number of residues in the shown protein domain.

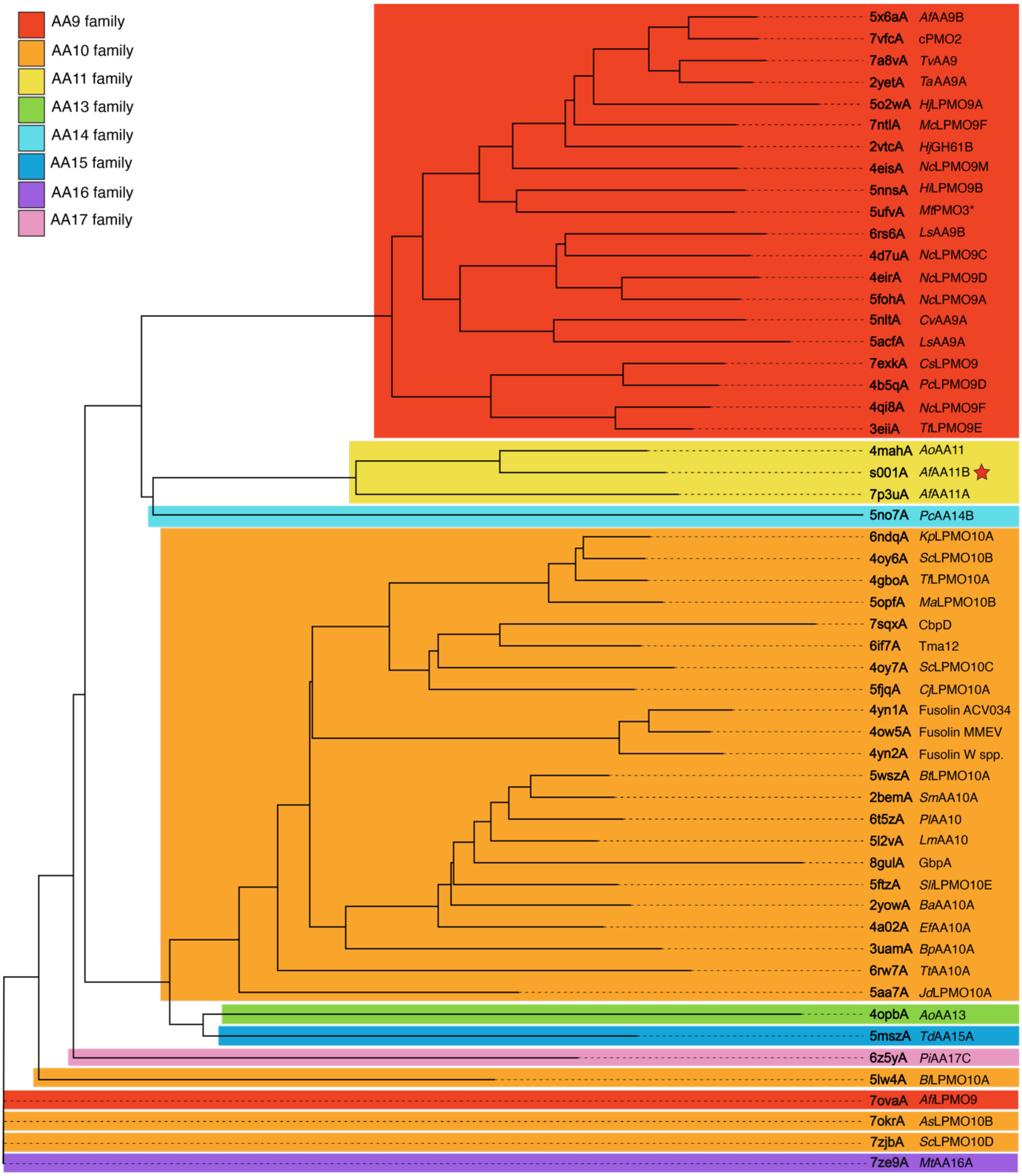

Figure S5. Dendrogram showing structural clustering of LPMO structures. Structural alignment, clustering and generation of the dendrogram was performed using the all-against-all-structure-comparison tool on the Dali server[2] for *Af*AA11B and other structures for the auxiliary activity families 9-11 and 13-17 (n = 53). Structures are identified by their PDB code and the chain ID, followed by the LPMO name listed in the CAZy database[3]. The different LPMOs are coloured according to their respective families. *Af*AA11B, which is the subject of this study, is indicated by a red star.

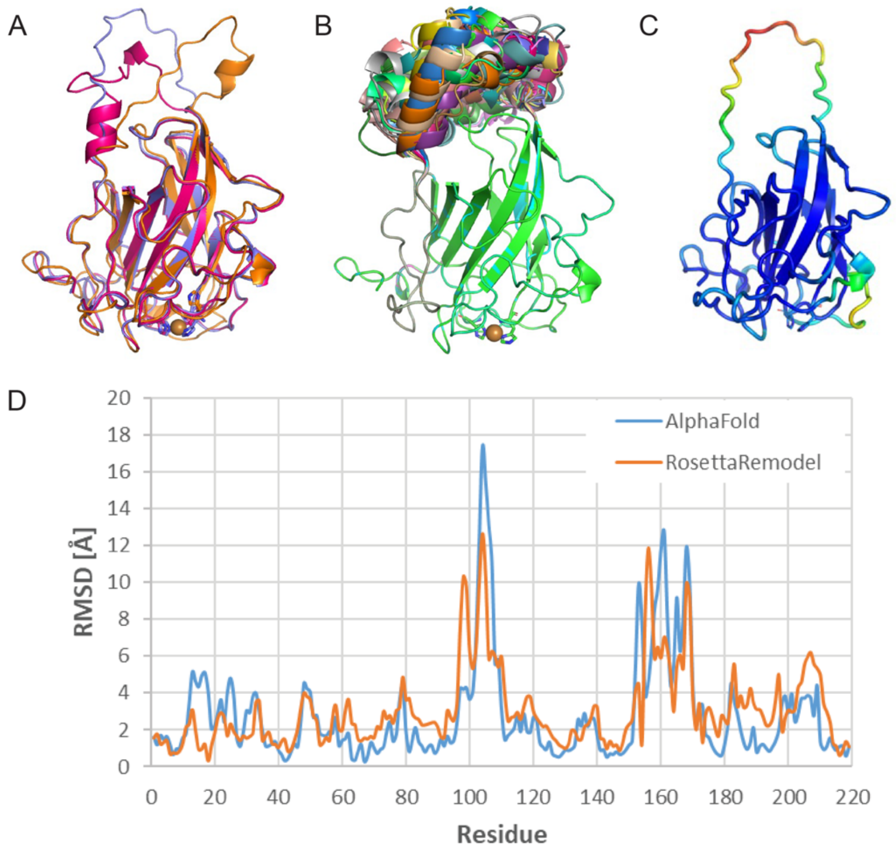

Figure S6. Molecular models of *Af*AA11B with reconstructed loop 152-172. (A) Superposition of the best models obtained from AlphaFold (purple), RoseTTAFold (orange) and SwissModel (pink). All the three methods suggest a largely disordered loop. (B) Superposition of the best 100 models generated by RosettaRemodel. Despite the large differences in the configuration of the loop, models include the presence of alpha helices in the residue range 152-172. (C) Structure at the end of the 1 μs MD simulation initialised with the highest ranking RosettaRemodel-generated structure, showing the loss of the helical structures. The protein is colour-coded by RMSF (blue stable regions, red unstable regions), highlighting that loop 152-172 is a highly disordered and flexible region. Loops 12-16 and 95-110 are also observed as flexible regions of *Af*AA11B. (D) Per-residue RMSD between the final frame of the 1 μs MD simulation and the starting structure, calculated for the AfA11B models generated via AlphaFold and RosettaRemodel. Both models show a high mobility of regions 95-110 and 152-172, matching the RMSF analysis.

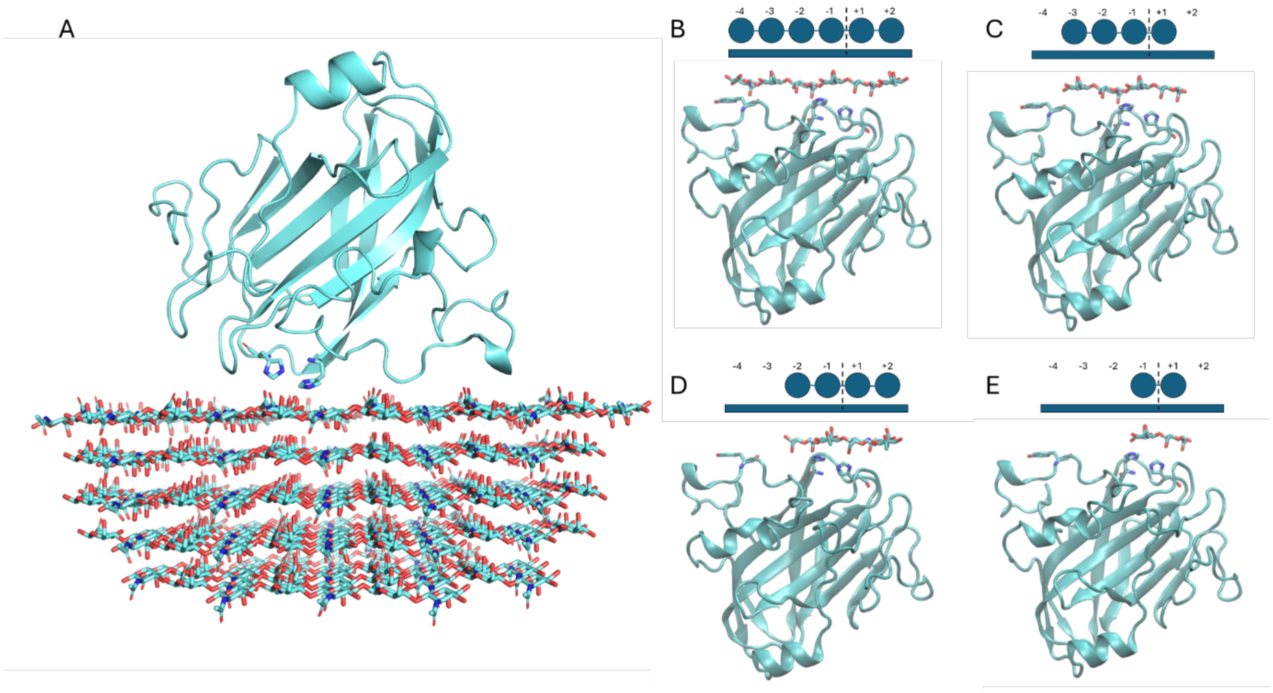

Figure S7. **Initial molecular models of *Af*AA11A in complex with crystalline β-chitin and chitin oligomers.** (A) Model of crystalline β-chitin in complex with *Af*AA11A, generated starting from the model described by Bissaro *et al.*[4,5] and by aligning the histidine brace of *Af*AA11A with the one of *Sm*AA10A (the LPMO present in the original model). (B-E) Models of *Af*AA11A in complex with oligomers ranging from hexamer (B), tetramers (C-D) and dimers (E) were generated by trimming the crystalline chitin in the model shown in panel A, leaving only part of the chitin chain that is binding to the catalytic copper site. Tetramers have been modelled by considering two different placements, binding from subsite −3 to +1 (C) and from subsite −2 to +2 (D). The dashed line between sugar units −1 and +1 indicates the cleavage site. Similar models were made for *Af*AA11B. Note that the tyrosine residue interacting with the sugar bound in the −4 subsite (panel B) is lacking in *Af*AA11B (see also Figure 2B of the main manuscript). Also note that, while the copper is not shown in the figures, it was included in the simulations.

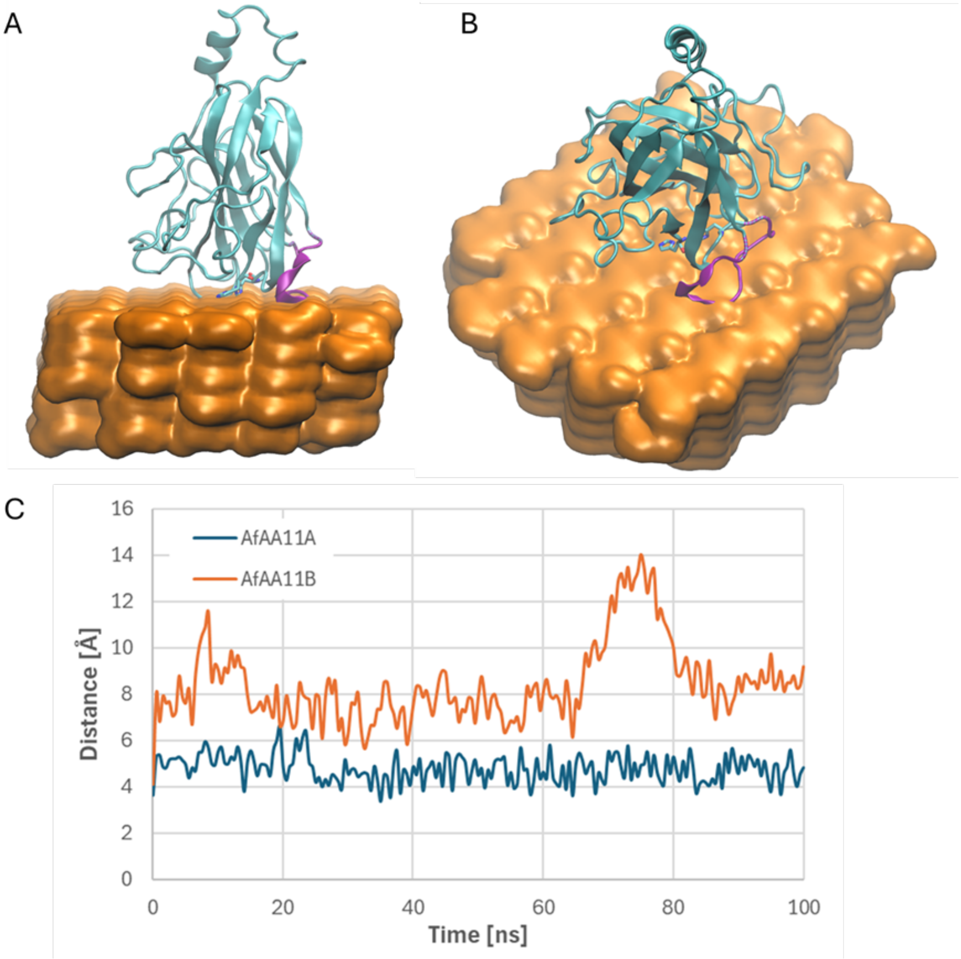

Figure S8. **Molecular dynamics simulation of *Af*AA11B in complex with crystalline chitin.** The initial structure of *Af*AA11B in complex with crystalline chitin (A-B) was generated by aligning the histidine brace of *Af*AA11B with the histidine brace of *Sm*AA10A (CBP21) present in the model described by Bissaro *et al.*[4,5], as explained in the Materials and Methods section. In this initial configuration, the average distance between the histidine brace and the chitin surface is 4.3 Å, and there are significant clashes between loop 95-110 (shown in purple colour) and the chitin surface, as highlighted by the fact that the loop disappears below the chitin surface (B). While the copper is not shown in the figures, it was included in the simulations. The distance between the histidine brace and the chitin is defined as the distance between the copper and the closest chitin atom (excluding hydrogen atoms). The distance plot shows that the copper site immediately moves away from the chitin. In the last 50 ns of the MD simulation, the average distance between the histidine brace and the chitin surface was 9.0 ± 1.2 Å. In a similar simulation with *Af*AA11A, the protein remained stably bound to the chitin and the average distance between the histidine brace and the chitin in the last 50 ns of the simulation was 4.6 ± 0.3 Å (panel C).

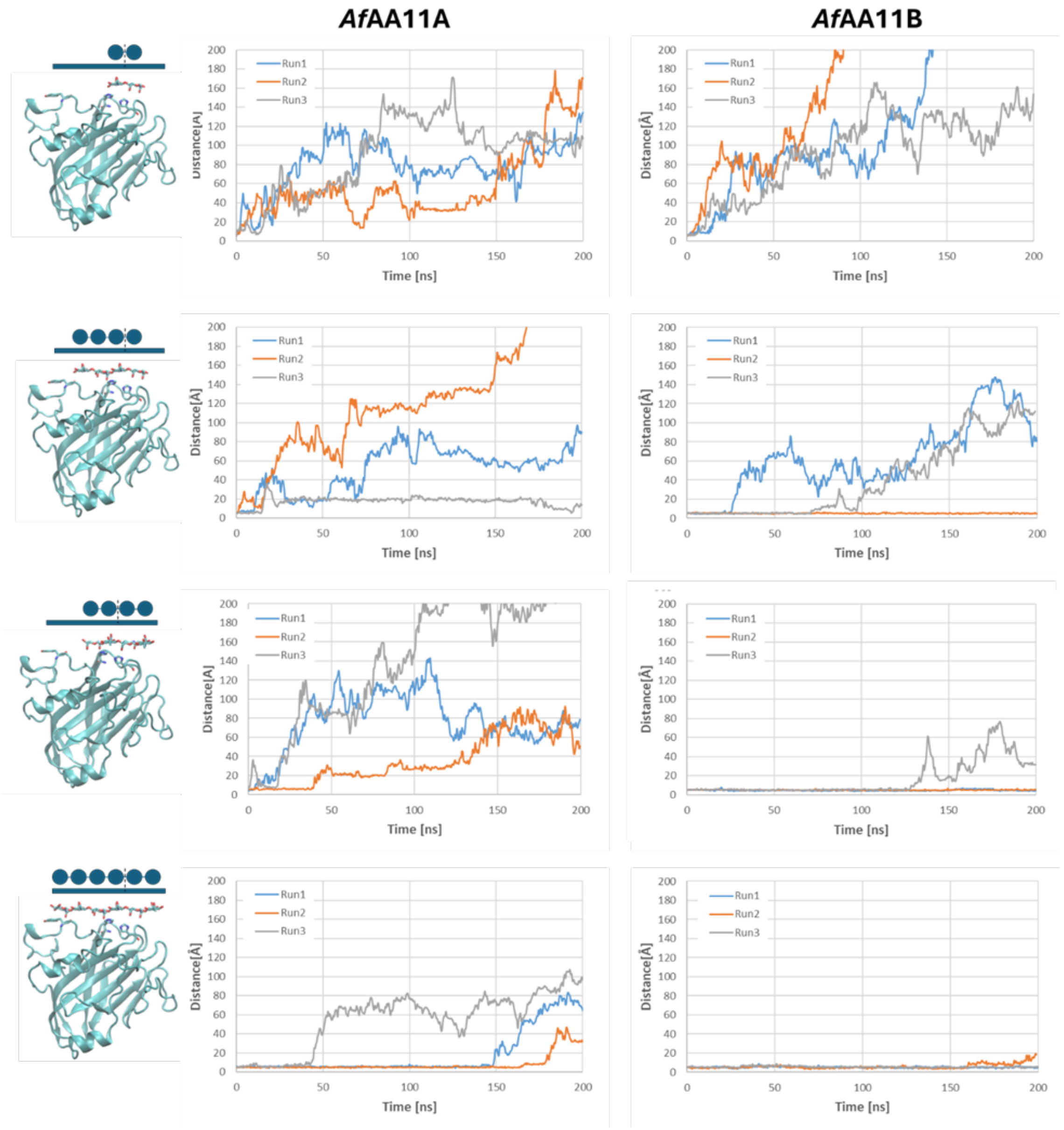

Figure S9. Distance between chitin oligomers and the histidine brace of *Af*AA11A (left) and *Af*AA11B (right) as observed during 200 ns MD simulations. Information on the starting models is provided in Figure S7. For each set-up, three runs are shown. The data show that oligomers quickly unbind from *Af*AA11A, with the partial exception of one of the tetramer runs (subsite -3 to +1) and the hexamer runs, where the bound position persists for longer. In the case of *Af*AA11B, the chitin dimer and chitotetraose when bound in subsites –3 to +1 quickly unbind from the protein, whereas chitotetraose bound in subsite –2 to +2 and chitohexaose mostly retain the bound position. While the copper is not shown in the figures, it was included in the simulations. The distance between the histidine brace and the oligomer is defined as the distance between the copper and the center of mass of the oligomer.

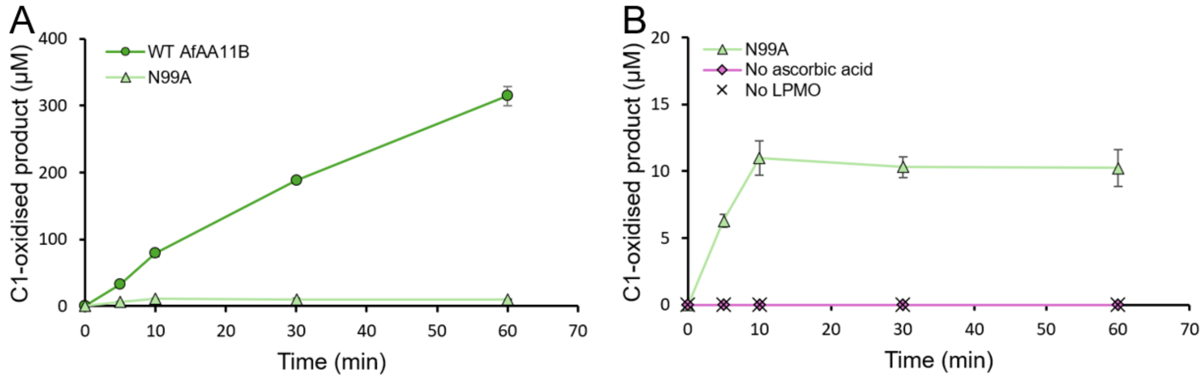

Figure S10. Activity of the N99A mutant of AfAA11B on chitotetraose. (A) Formation of C1-oxidised product by the wild-type enzyme and the N99A mutant over 60 min. (B) A close-up of product formation for the N99A mutant presented in A, as well as the results for control reactions without LPMO and without reductant. The mutant appears to rapidly inactivate, as one would expect when binding to the substrate is weak, promoting damaging off-pathway peroxidase reactions. All reactions contained 1 µM LPMO, 1 mM $(GlcNAc)_4$, and 1 mM ascorbate in 50 mM Bis-Tris, pH 6.5, and were performed in a thermomixer at 37 °C, 800 rpm. Data reported for *Af*AA11B WT is the average of 2 independent measurements. Data for the N99A mutant is the average of 3 independent measurements. Error bars are standard deviations and are hidden by the symbols in some cases. The oxidase activity of the N99A mutant at pH 6.5 was determined to be 0.12 ± 0.01 s^{-1}, which is not significantly different from the oxidase activity recorded for the wild-type (Fig. 5B of the main manuscript), implying that the N99A mutant is folded and binds copper.

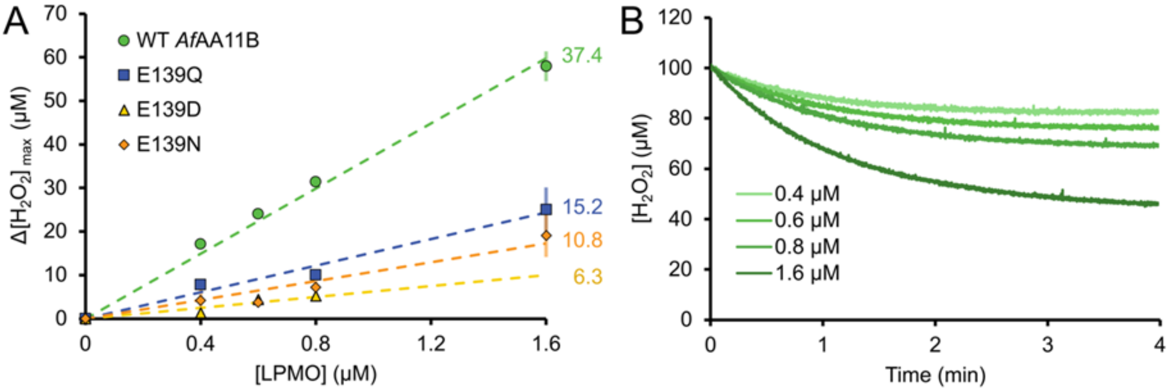

Figure S11. Impact of mutating Glu139 on inactivation of *Af*AA11B in the ascorbate peroxidase reaction. (A) Linear relationship between the maximum amount of H_2O_2 turned over and the concentration of LPMO in the reductant peroxidase reaction, monitored using an electrochemical H_2O_2 sensor. Error bars are standard deviations and are hidden by the symbols in some cases; data is at least duplicates, except in the case of E139D, where only one measurement for each concentration of LPMO was made. Dotted lines show linear regression of the data with an intercept set at 0. The value of the slope of each line is shown in the graph and represents the average number of peroxidase reactions the LPMO variant can catalyse before inactivation[6]. (B) Example progress curves for WT *Af*AA11B underlying the data presented in A. Only single experiments are shown for the sake of clarity. Each curve was fit using non-linear regression according to the following equation: $[H_2O_2] = \Delta[H_2O_2]_{max}e^{-k^{app}t} + [H_2O_2]_\infty$, where $\Delta[H_2O_2]_{max}$ is the maximum amount of H_2O_2 turned over, k^{app} is the apparent first order rate constant of H_2O_2 consumption and $[H_2O_2]_\infty$ is the final H_2O_2 concentration[6]. R^2 was over 0.99 for all curves. All reactions contained 100 μM H_2O_2 and were initiated by the addition of 200 μM ascorbate. Control reactions without ascorbate or without LPMO showed no detectable H_2O_2 consumption over four minutes (not shown). Reactions were monitored using a rotating disk electrode with an angular velocity of 50 s^{-1}, at 30 °C in 50 mM Bis-Tris, pH 6.5, 100 mM KCl and 5 μM EDTA. A low concentration of EDTA (insufficient to deplete copper from the LPMO on this timescale) was added in these reactions to scavenge free copper released from damaged LPMO molecules[6].

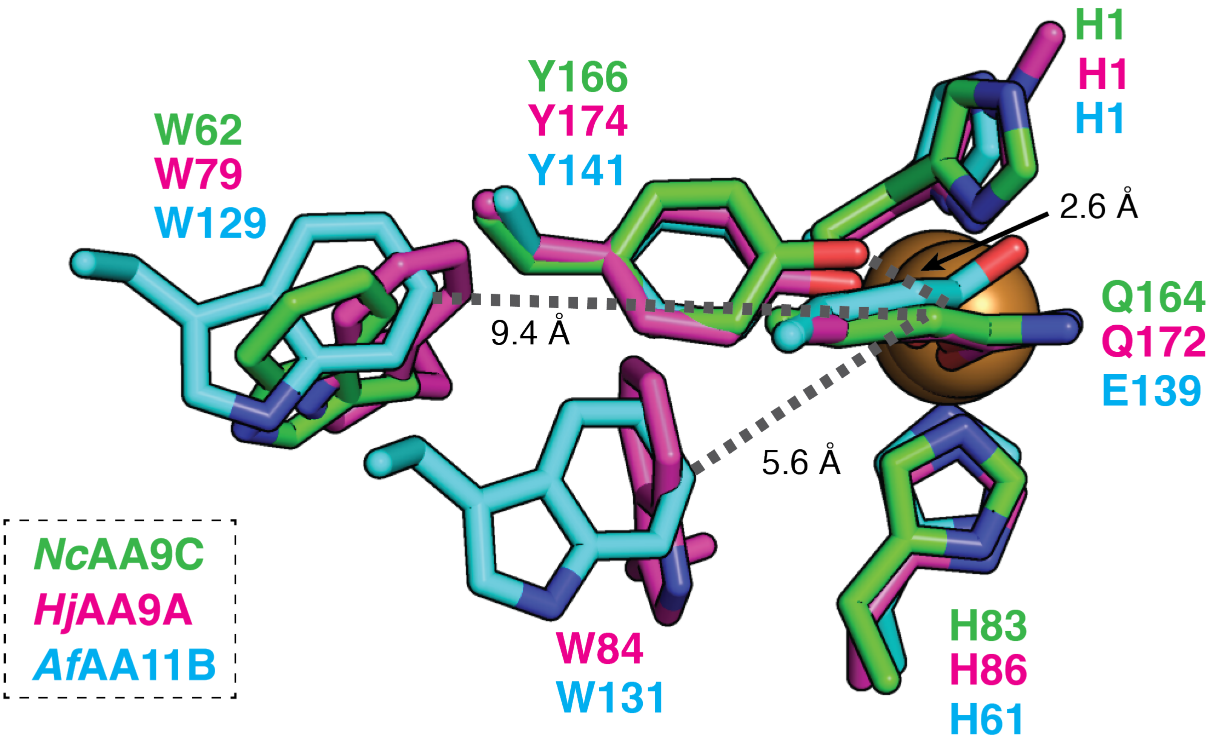

Figure S12. Structural alignment of *Af*AA11B, *Nc*AA9C and *Hj*AA9A. Tyrosine and tryptophan residues close to the copper are shown. The histidine brace, the conserved Glu/Gln residue and the copper ion are also shown. *Nc*AA9C (PDB: 4D7U, green), *Hj*AA9A (PDB: 5O2W, pink) and *Af*AA11B (blue) were aligned using the alignment feature in PyMOL. The distance between the copper ion and W131, W129 or Y141 in *Af*AA11B is indicated by the dashed grey lines with the distance reported in Å. Note that H1 is methylated in *Hj*AA9A.

Table S1. List of primers used for cloning the *Af*AA11B variants.

Primer pair	Primer name	Sequence in 5′→ 3′
1	pBSY3Z-FWD	ATGAGAAGAGCGAATTCGGC
	pBSY3Z-REV	ATTTAAATACATGTGAGCAAAAGGCCAG
2	P_{DAS2}-3Z-FWD	TGCTGGCCTTTTGCTCACATGTATTTAAATCTATGCTACCCCACA GAAATAC
	P_{DAS2}-3Z-REV	TTACCGGCGCGCCGAATTCGCTCTTCTCATTTTTGATGTTTGATAG TTTG
3	*AOX1*TT-FWD	GCGGCCGCTCAAGAGGATGTCAG
	P_{DAS2}-REV	TTTTGATGTTTGATAGTTTGATAAG
4	*Af*AA11B-FWD	TCACTCTTATCAAACTATCAAACATCAAAAATGAGACAGGTCTG GTTCTCTTG
	*Af*AA11B-REV	TGGCATTCTGACATCCTCTTGAGCGGCCGCTTAAGCGTGTCTGTG TCCACC
5	*Af*AA11B-E160D-FWD	CGGTAATTGGAGCACAGTTCATGTACATGTCTCTGTTACCGATTC TGTTG
	*Af*AA11BGate-REV*	ATGTACATGAACTGTGCTC
6	*Af*AA11B-E160N-FWD	CGGTAATTGGAGCACAGTTCATGTACATGTTTCTGTTACCGATTC TGTTG
	*Af*AA11BGate-REV*	ATGTACATGAACTGTGCTC
7	*Af*AA11B-E160Q-FWD	CGGTAATTGGAGCACAGTTCATGTACATTTGTCTGTTACCGATTC TGTTG
	*Af*AA11BGate-REV*	ATGTACATGAACTGTGCTC
8	*Af*AA11B-N99A-FWD	GTTGACGGTGCTATGTCTGGTGGTGCCGACGTCCCAGAC
	*Af*AA11B-N99A-REV	AGACATAGCACCGTCAACGTTAGCAGGACAACCACCTTC
9	*Af*AA11B-short-FWD	TAACTTACAACCAGCTGGTGAAGCCGCTTGTTAAGCGGCCGCTC AAGAGGATGTC
	*Af*AA11B-short-REV	TGGCATTCTGACATCCTCTTGAGCGGCCGCTTAACAAGCGGCTTC ACCAGCTGG

*Primers are identical

3. Supplementary References

1) Eisenberg, D.; Schwarz, E.; Komaromy, M.; Wall, R. *J Mol Biol* **1984**, *179*, 125-142.
2) Holm, L.; Rosenstrom, P. *Nucleic Acids Res.* **2010**, *38*, W545-549.
3) Drula, E.; Garron, M. L.; Dogan, S.; Lombard, V.; Henrissat, B.; Terrapon, N. *Nucleic Acids Res.* **2022**, *50*, D571-D577.
4) Bissaro, B.; Isaksen, I.; Vaaje-Kolstad, G.; Eijsink, V. G. H.; Røhr, Å. K. *Biochem.* **2018**, *57*, 1893-1906.
5) Bissaro, B.; Streit, B.; Isaksen, I.; Eijsink, V. G. H.; Beckham, G. T.; DuBois, J. L.; Røhr, Å. K. Molecular mechanism of the chitinolytic peroxygenase reaction. *Proc. Natl. Acad. Sci. USA* **2020**, *117*, 1504-1513.
6) Kuusk S.; Eijsink, V. G. H.; Väljamäe P. The "life-span" of lytic polysaccharide monooxygenases (LPMOs) correlates to the number of turnovers in the reductant peroxidase reaction. *J. Biol. Chem.* **2023**, *299*, 105094.
